# Dominance and leadership in research activities: Collaboration between countries of differing human development is reflected through authorship order and designation as corresponding authors in scientific publications

**DOI:** 10.1371/journal.pone.0182513

**Published:** 2017-08-08

**Authors:** Gregorio González-Alcaide, Jinseo Park, Charles Huamaní, José M. Ramos

**Affiliations:** 1 Department of History of Science and Documentation, University of Valencia, Valencia, Spain; 2 Korea Institute of Science and Technology Information (KISTI), Daejeon, South Korea; 3 Servicio de Neurología, Hospital Nacional Guillermo Almenara, La Victoria, Perú; 4 Department of Clinical Medicine, Miguel Hernández University of Elche de Elche, Alicante, Spain; 5 Department of Internal Medicine, Hospital General Universitario de Alicante, Alicante, Spain; Centre for Research and Technology-Hellas, GREECE

## Abstract

**Introduction:**

Scientific collaboration is an important mechanism that enables the integration of the least developed countries into research activities. In the present study, we use the order of author signatures and addresses for correspondence in scientific publications as variables to analyze the interactions between countries of very high (VHHD), high (HHD), medium (MHD), and low human development (LHD).

**Methodology:**

We identified all documents published between 2011 and 2015 in journals included in the Science Citation Index-Expanded categories’ of Tropical Medicine, Infectious Diseases, Parasitology, and Pediatrics. We then classified the countries participating in the publications according to their Human Development Index (HDI), analyzing the international collaboration; positioning and influence of some countries over others in cooperative networks; their leadership; and the impact of the work based on the HDI and the type of collaboration.

**Results:**

We observed a high degree of international collaboration in all the areas analyzed, in the case of both LHD and MHD countries. We identified numerous cooperative links between VHHD countries and MHD/LHD countries, reflecting the fact that cooperative links are an important mechanism for integrating research activities into the latter. The countries with large emerging economies, such as Brazil and China stand out due to the dominance they exert in the collaborations established with the United States, the UK, and other European countries. The analysis of the leadership role of the countries, measured by the frequency of lead authorships, shows limited participation by MHD/LHD countries. This reduced participation among less developed countries is further accentuated by their limited presence in the addresses for correspondence. We observed significant statistical differences in the degree of citation according to the HDI of the participating countries.

**Conclusions:**

The order of signatures and the address for correspondence in scientific publications are bibliographic characteristics that facilitate a precise, in-depth analysis of cooperative practices and their associations with concepts like dominance or leadership. This is useful to monitor the existing balance in research participation in health research publications.

## Introduction

Numerous studies and reports have warned that medical initiatives and research programs in low-income countries are continually underfunded and underrepresented [1–3], which is evidenced by the limited contributions of these countries to the mainstream, highly cited scientific journals that give visibility to priority topics among the research community [4,5].

Authorships in scientific publications are the mechanism through which scientists assume responsibility for published content and take credit for new ideas or discoveries. Quantifying authorship characteristics enables the analysis of researchers’ contributions toward the development of knowledge in a discipline; and by extension of the institutions, countries, and geographic regions to which they are attributed [6]. The International Committee of Medical Journal Editors (ICMJE) establishes a set of criteria for determining when an author should sign and assume responsibility for research papers published in medical journals [7]. In spite of the criticism received for these criteria [8,9] and the authorship inflation reported by some studies as evidence that the ICMJE guidelines are not rigorously followed [10,11], these criteria establish the framework that has become the leading standard in health science research to determine who is an author. But when more than one author is involved in a paper, the question of author position arises. Rules for the order of multiple authors in a document are generally consistent within a field. In the health sciences, authorship is often attributed in descending order of contribution: the first author is the main contributor in terms of involvement and/or leadership in the work, while the contributions of subsequent authors have successively less weight. The corresponding authors and final authors are also considered to have a prominent role relative to the other signatories of the publication, positions associated in this case with the assumption of responsibility in the direction of work and the published contents [12–17].

Signature order and the address for correspondence may constitute, therefore, bibliographic characteristics that can be captured in the form of indicators to measure the role played by authors and their degree of contribution to research in a given discipline or area [18,19]. Likewise, in papers with international collaboration, studying the order of signatures and participation as corresponding authors may also provide information about dominance and leadership in research, shedding light on North-South relationships in countries participating in research activities [20,21].

Numerous studies have used the order of author signatures in health sciences publications to analyze the existence of a gender gap in research activities [22,23]. Occasionally, investigators have also used signature order to quantify the productivity of institutions or individual contributions of researchers within collaborative papers [24]. Other authors have called for this variable to be considered when calculating citation indicators in order to acknowledge the merit of each author participating in the papers [25–27]. Although some studies that analyze the participation of less developed countries in research activities do provide data on their researchers’ contribution as first authors [4,28,29], we were not able to identify any studies that specifically focus their research question on this aspect or discuss its implications.

The aim of the present study is to analyze the order of signatures and the addresses for correspondence in order to determine their utility as variables for monitoring the existing interactions between countries with differing levels of human development. We use these indicators to explore the cooperative and citation practices in scientific publications in four research areas that are of special relevance to less developed countries: Tropical Medicine, Infectious Diseases, Parasitology, and Pediatrics.

## Methods

The methodological process we carried out included the following elements.

### Performance of bibliographic searches to identify the group of documents under study

We performed a bibliographic search in the Science Citation Index-Expanded database (SCI-Expanded), identifying all articles and reviews published between 2011 and 2015 in the categories of Tropical Medicine, Infectious Diseases, Parasitology, and Pediatrics. We selected these categories because of their special relevance to less developed countries. Infectious and parasitic diseases disproportionately affect these regions, while Tropical Medicine is of special interest because many developing countries are located in the tropics, where the climatic conditions exist for the development of these specific—often considered neglected—diseases. Finally, as regards pediatrics, less developed countries have the highest rates of both stillbirth and infant mortality, so basic and clinical research on diseases affecting the pre-adult population should also be a priority. As an example, the World Health Organization (WHO) noted that communicable diseases, maternal causes, and nutritional deficiencies caused over half of all deaths in low-income countries in 2015, while these fewer than 7% of deaths were attributable to such causes in high-income countries [30–33]. We selected the SCI-Expanded database of the Web of Science (WoS) because it is the main multidisciplinary database at an international level that brings together the mainstream journals of reference for their visibility and impact.

### Downloading of the bibliographic characteristics of the documents, identification of the participating countries and standardization of data

We downloaded the information contained in the fields for institutional affiliation (field C1 in the WoS) and corresponding author (field RP), identifying the countries and geographic regions mentioned in the specified fields. Nearly all (99.34%) of the documents we analyzed had institutional affiliations, and 99.33% had addresses for correspondence. We standardized the data retrieved, unifying institutional affiliations for England, Northern Ireland, Scotland and Wales as the United Kingdom (UK). Overseas French and British territories and islands without their own internationally recognized political entities were assigned to their corresponding country (documents signed by authors in French Polynesia, Guadeloupe, Martinique, New Caledonia, and Reunion were assigned to France, although we did maintain their geographic links to the corresponding region). Other bibliographic characteristics of the documents used in the study were the number of citations received by the documents (field TC) and the year of publication (PY).

### Categorization of countries according to geographic and human development criteria

We classified countries responsible for publications according to their Human Development Index (HDI) into very high human development (VHHD), high human development (HHD), medium human development (MHD), and low human development (LHD). The HDI is a measure published by the United Nations Development Programme of average achievement in key dimensions of human development. The population distribution of the countries analyzed for their HDI is roughly balanced between countries of high/very high human development (54% of the global population) and those with medium/low human development (46%) (S1 Table). In addition, we assigned each of the documents to one of the categories detailed in Table 1, which combines the HDI of the countries and the order or position occupied by the signing authors from those countries.

**Table 1:** Collaboration types considering countries HDI and the order of signatures in scientific publications.

Type	First position	Second and subsequent positions	Description
1	VHHD/HHD	-	Collaboration within a single country of very high or high human development
2	VHHD/HHD	VHHD/HHD	Collaboration between two or more countries of very high or high human development
3	MHD/LHD	-	A single country of medium or low human development
4	MHD/LHD	MHD/LHD	Collaboration between two or more countries of medium or low human development
5	VHHD/HHD	MHD/LHD	Leadership of a country of very high or high human development and participation of one or more countries of medium or low human development
6	MHD/LHD	VHHD/HHD	Leadership of a country of medium or low human development and participation of one or more countries of very high or high human development
7	VHHD/HHD	VHHD/HHD + MHD/LHD	Leadership of a country of very high or high human development with simultaneous participation of other countries of very high or high human development and medium or low human development
8	MHD/LHD	MHD/LHD + VHHD/HHD	Leadership of a country of medium or low human development with simultaneous participation of other countries of medium or low human growth and high or very high human development

VHHD: very high human development; HHD: high human development; MHD: medium human development; LHD: low human development.

Each of the countries identified was assigned to a macro geographic (continental) region according to the groups established by the United Nations Statistics Division and presented in S2 Table [34]. Asia is the region that concentrates the highest density population (about 60% of the total), followed at some distance by Africa (14%), Europe (11%), Latin America and the Caribbean (8%), North America (5%), and Oceania (0.5%). We observed only small variations with regard to the number of countries participating in the research activity of each discipline analyzed (S2 Table). S1 Appendix presents the distribution, HDI and geographic region of all countries identified.

### Indicators obtained and concepts used in the study

We calculated the absolute number of participations in the documents by country, geographic region and HDI. For example, a document co-authored by three researchers from the United States (North American VHHD country), two from Kenya and one from Lesotho (both African LHD countries) counts as a single paper in each country’s total, a single paper for both North America and Africa, a single paper for both VHHD and LHD countries, and as one link between each country pair.

Once the above steps were complete, we analyzed the following aspects.

#### International collaboration, position and influence within the cooperative networks

Our initial approach to the topic involved determining the percentage of documents signed jointly by two or more countries, which is a widely used indicator to analyze cooperative practices. In addition to the geographic areas indicated, we specifically analyzed the international collaboration involving reference countries for each region in order to establish whether differences existed between the degree of collaboration observed for those countries and the overall degree of collaboration observed for their respective regions. In the case of Africa, we selected Nigeria (the most densely populated country and a reference for the predominantly LHD countries on the continent) and South Africa (MHD country that is a prominent reference because of its size and the impetus given to developing its research system in recent years). The situation in Asia is more heterogeneous, with countries of all HD categories; we selected the two countries comprising the largest population: China (HHD) and India (MHD), along with Japan (VHHD) because of its importance among VHHD countries and Pakistan because it is the most densely populated LHD country. Most countries in Europe fall into the VHHD category, so we chose the three most populated countries with the greatest scientific development (the UK, Germany, and France) in order to assess any significant differences with relation to the rest of the countries in the region. In North America, we selected the United States (VHHD); in Oceania, Australia (VHHD); and in Latin America and the Caribbean, Brazil (HHD), as these are the reference countries in their respective regions due to their size, population, and scientific development.

We generated a collaboration network among the countries for each of the four areas of knowledge in order to analyze the position and collaborative relationships existing between the countries according to the level of human development. In this undirected network, the size of the nodes was proportional to the number of documents, and the distance and thickness of links between them reflected the strength of the collaborative relationship (a thicker link with a smaller distance indicates a stronger relation). The color of the nodes represents the countries’ HDI (red VHHD, green HHD, blue MHD, yellow LHD). We used Gephi software for generating the networks; to facilitate visualization and interpretation, we limited the graphics to the top 300 links. To analyze the relative position of the different countries in the networks and the distribution of their collaborative interactions, we calculated the following indicators: centrality, degree, and number of collaborative links to countries pertaining to the different HDI categories.

We also measured the influence exercised by some countries over others with regard to the cooperative activities that they carried out together. The concept of *influence* in the collaboration networks is defined as the predominance, authority, or dominant role of one country over another. In collaborative papers, the first author (and by extension their institution or country) presumably assumes more responsibility in the research. In order to integrate the concept of influence into the analyses of international collaboration and collaboration networks between countries, we first quantified the order of signatures in the documents signed in collaboration, calculating a *dominance index* between each country pair. We then constructed a directed network to represent the influence exercised by one country over another, as follows: First, the direction of arrows between two countries (*i*, *j*) is determined by considering the weights of (*i*, *j*) and (*j*, *i*) collaborations. We considered that the country appearing most frequently under the first author’s affiliation wielded a larger influence in the research collaborations existing between them, which is reflected by the direction of the arrows in the network links. For example, in the case of Brazil and the United States and in the area of Tropical Medicine, the direction is Brazil → USA, as Brazil has a larger presence in the lead author position among the documents signed jointly between the two countries (n = 200 versus n = 71). Second, the weight of the line is expressed by (*i*, *j*) + (*j*, *i*). In the case of Brazil and the USA, the weight is 271. For ease of visualization, we excluded links with a weight of less than 15. Third, the label of the line represents the numerical expression of the dominance index between two countries. This is defined as max {(i, j), (j, i)} / {(i, j)+(j, i)}. In the case of Brazil and the USA, the dominance index is max {(200, 71)} / {(200 + 71) = 0.74. The dominance index ranges from 0.5 to 1, with values closer to 1 indicating a greater influence of one country over another. For example, in the case of Germany and Burkina Faso, authors from Germany led 14 documents in Tropical Medicine written in collaboration with authors from Burkina Faso, while authors from Burkina Faso did not hold the lead authorship in any, so in this case the dominance index Germany → Burkina Faso is 1.

To illustrate the process described and the interest of the proposed indicator, the results section presents the analysis performed in the category of Tropical Medicine, as this is the area showing the greatest participation from MHD and LHD countries. S3 Table and S1 Fig (Infectious Diseases), S4 Table and S2 Fig (Parasitology) and S5 Table and S3 Fig (Pediatrics) show the results for the rest of the categories.

#### Contribution and leadership in research activities

Quantifying the number of documents published and their weight relative to the total scientific production in each area of knowledge generates an indicator of the *contribution* of the country groups according to both their HDI and their geographic region.

The concept of *leadership* that we propose in the present study is intended to deepen and complement the information provided by the analysis of scientific contributions, assigning a different role or weight to each. *Leadership* in research activity may be defined as the degree to which the author (or country) assumes responsibility for directing the scientific work being developed. We understand that it is possible to obtain an approximation of the concept of leadership in the area of biomedicine based on bibliographic analysis and by quantifying authors’ (or their countries’) participation as first and corresponding author. To this end, we determined the percentage of documents with first authors and corresponding authors from each group of countries based on HDI, considering the overall document group as well as only the documents signed in collaboration, in order to differentially analyze both the general leadership associated with greater scientific contributions to the field of study, and the specific leadership in collaborative papers.

#### Impact of papers based on leadership and type of collaboration

Finally, we analyzed the degree of citation in the included papers, tying it to the concept of leadership and the types of collaboration. We first calculated the average degree of citations per paper, considering the different types of collaborations described in Table 1 for each of the four subject areas analyzed. For the statistical comparison of the average degree of citations per document according to each type of collaboration, we used the student’s *t* test. For a detailed comparison of the differences in citation degree between the document groups analyzed, we applied the one-way analysis of variance (ANOVA) using SPSS software (version 22.0). We also used Tukey’s HSD post hoc test when the null hypothesis in ANOVA was rejected to determine differences between the studied groups (collaboration types and categories), establishing a significance level of 5%.

All of the data used to carry out the study, including the information downloaded from the database as well as that derived from the treatment of the bibliographic entries, were deposited in the open access public repository, the Dataverse Project (https://dataverse.harvard.edu/, doi:10.7910/DVN/J51WO4).

## Results

### International collaboration, positioning, and influence in cooperative networks

**Fig 1:**
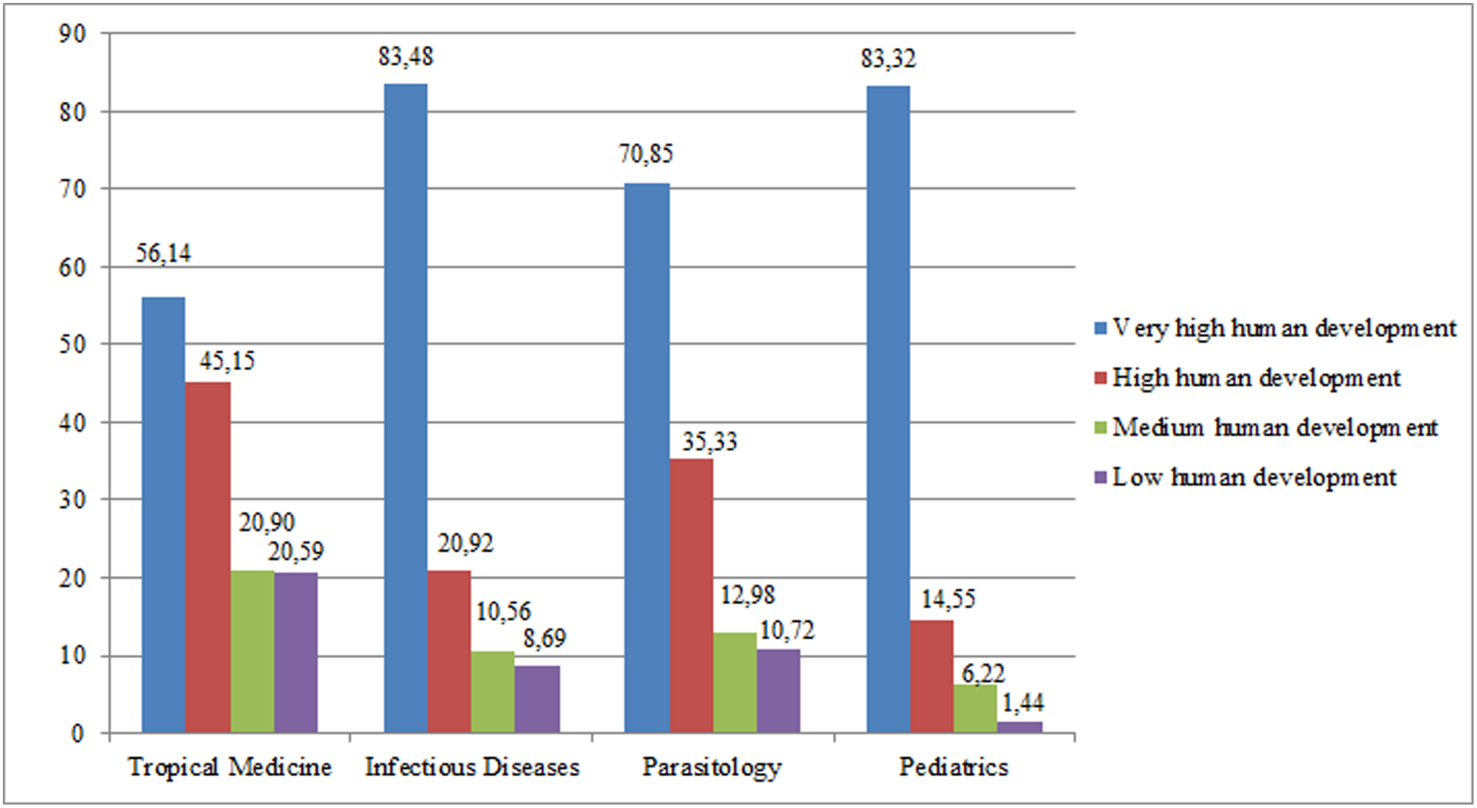
Degree of participation in the scientific publications (% of documents) according to the HDI of participating countries in the documents collected in SCI-Expanded in the categories of Tropical Medicine, Infectious Diseases, Parasitology and Pediatrics (2011–2015).

We included 186,756 documents published between 2011 and 2015 corresponding to the categories Tropical Medicine (N = 16,303), Infectious Diseases (N = 65,093), Parasitology (N = 28,606), and Pediatrics (N = 76,754). The scientific production of the different countries according to the HDI (Fig 1) shows that VHHD countries made the greatest contribution to the research, with participation values ranging from 56.14% of the documents in the case of Tropical Medicine to 83.48% in Infectious Diseases, in contrast with the reduced participation of MHD and LHD countries (1.44% to 20.9%).

By geographic areas (Table 2), there are important differences between the four categories analyzed: Asia leads research in Tropical Medicine, with participation from its authors in 36.37% of the documents; Europe ranks first in Infectious Diseases (42.68%) and Parasitology (40.87%); and North America in Pediatrics (43.78%). Africa's most important participation is in the field of Tropical Medicine (23.26% of the documents), followed at a distance by its contributions in Parasitology (14.32%) and Infectious Diseases (11.89%). For its part, Latin America and the Caribbean stand out in relation to the areas of Tropical Medicine (representation in 27.96% of the total documents) and Parasitology (20.57%). The scientific contributions in the area of Pediatrics are concentrated in North America and Europe, with limited participation from the rest of regions.

**Table 2:** Distribution of scientific contributions and degree of international collaboration by geographic area and country in the documents collected in SCI-Expanded in the categories of Tropical Medicine, Infectious Diseases, Parasitology, and Pediatrics (2011–2015).

**Regions**	**Tropical Medicine**		**Infectious Diseases**		**Parasitology**		**Pediatrics**	
	**N docs**	**IC (%)**	**N docs**	**IC (%)**	**N docs**	**IC (%)**	**N docs**	**IC (%)**
Africa	3793	3160 (83.31)	7743	6631 (85.64)	4097	3352 (81.81)	2223	1123 (50.52)
Asia	5930	2391 (40.32)	16,843	6453 (38.31)	8181	3616 (44.2)	17,444	3165 (18.14)
Europe	5189	4375 (84.31)	27,782	13,561 (48.81)	11,691	7829 (66.97)	24,979	7250 (29.02)
Latin America and the Caribbean	4558	2853 (62.59)	5476	2771 (50.6)	5884	2186 (37.15)	3000	1023 (34.1)
Northern America	4141	3229 (77.98)	24,802	11,062 (44.6)	8106	4624 (57.04)	33,606	6384 (19)
Oceania	766	624 (81.46)	3495	2050 (58.65)	1687	1057 (62.65)	3883	1566 (40.33)
**Reference countries**	**N docs**	**IC (%)**	**N docs**	**IC (%)**	**N docs**	**IC (%)**	**N docs**	**IC (%)**
Australia	708	569 (80.37)	3204	1871 (58.39)	1496	942 (62.97)	3474	1412 (40.64)
Brazil	2861	683 (23.87)	2805	1247 (44.45)	3773	1120 (29.68)	1541	384 (24.92)
France	935	749 (80.11)	5147	2824 (54.87)	1954	1498 (76.66)	2702	874 (32.35)
Germany	489	447 (91.41)	3252	2105 (64.73)	1718	1282 (74.62)	3717	1474 (39.65)
India	1471	357 (24.27)	2120	871 (41.08)	1324	368 (27.79)	3067	424 (13.82)
Japan	330	274 (83.03)	2793	818 (29.29)	1008	666 (66.07)	2694	278 (10.32)
Nigeria	346	179 (51.73)	435	329 (75.63)	238	149 (62.6)	216	80 (37.04)
Pakistan	101	48 (47.52)	315	195 (61.9)	156	84 (53.85)	143	88 (61.54)
China	959	264 (27.53)	3996	1441 (36.06)	2076	768 (36.99)	2403	545 (22.68)
South Africa	349	263 (75.36)	2036	1655 (81.29)	559	432 (77.28)	550	306 (55.64)
UK	2127	1941 (91.25)	7560	5066 (67.01)	3515	2785 (79.23)	5838	2505 (42.91)
USA	3947	3086 (78.18)	22,788	10,378 (45.54)	7577	4349 (57.4)	30,113	5500 (18.26)
*Total (all countries)*	*16*,*240*	*7203 (44*.*35)*	*64*,*859*	*21*,*246 (32*.*76)*	*28*,*450*	*11*,*468 (40*.*31)*	*75*,*979*	*11*,*355 (14*.*94)*

N docs: Number of documents with institutional affiliations; IC: International collaboration.

**Fig 2:**
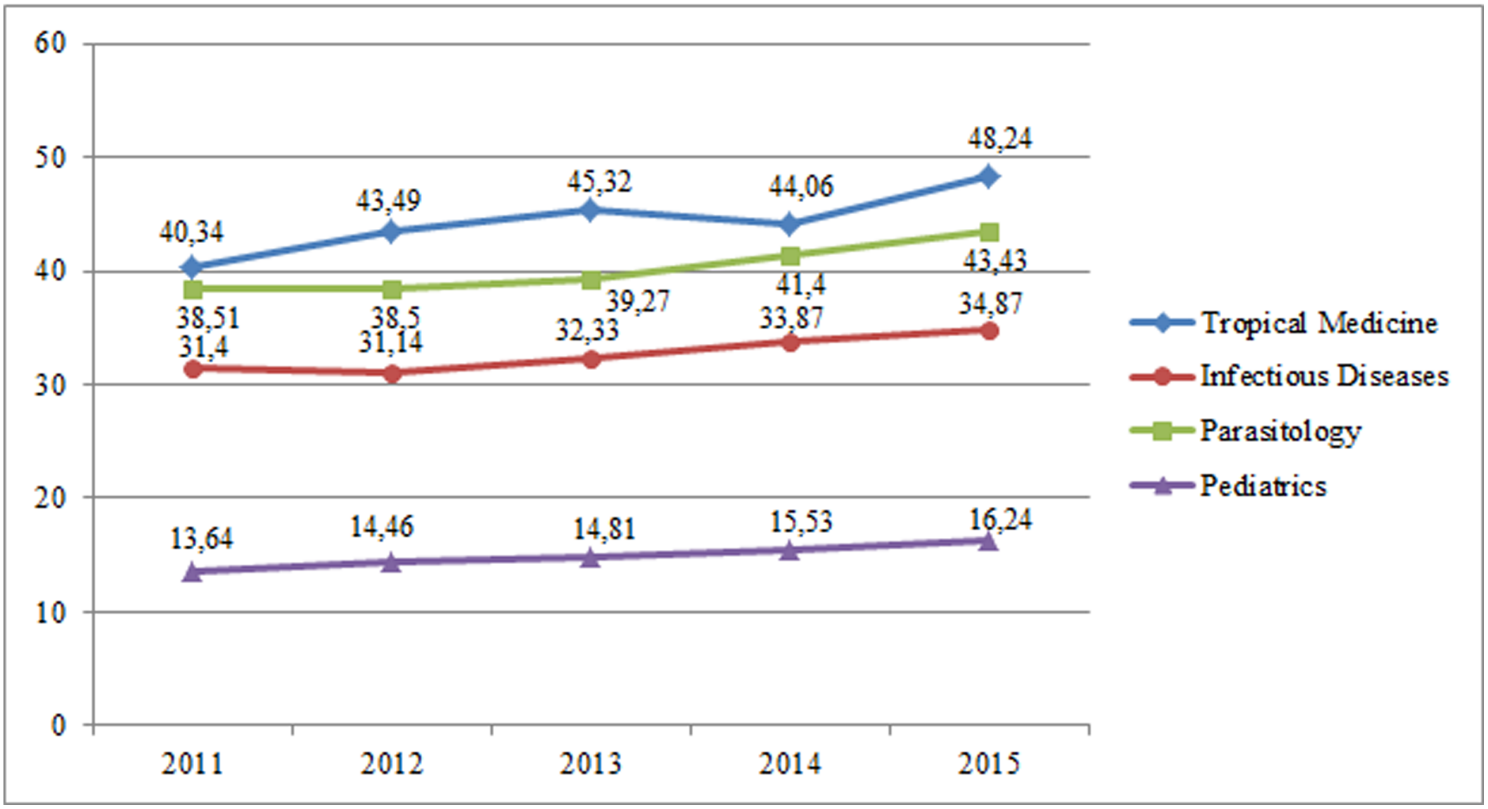
Percentage of documents signed in international collaboration in SCI-Expanded database in the categories of Tropical Medicine, Infectious Diseases, Parasitology, and Pediatrics (2011–2015).

We observed the highest level of international collaboration in Tropical Medicine (44.35% of the documents, n = 7203), followed by Parasitology (40.31%, n = 11,468), Infectious Diseases (32.76%, n = 21,246), and Pediatrics (14.94%, n = 11,355). There was a trend toward an increase in international collaboration over the study period (Fig 2); this was more pronounced in the case of Tropical Medicine, where 40.34% of the documents were signed in international collaboration in 2011 and 48.24% in 2015. The same occurred in Parasitology, where the proportion of international collaborations increased from 38.51% in 2011 to 43.43% in 2015.

The most significant aspect of the analysis of international collaboration according to country HDI (Table 3) was the elevated degree of collaboration observed in the case of LHD countries, which stood around 70.41% of all documents in Pediatrics and 88.81% in Infectious Diseases. MHD countries also presented a high degree of collaboration (26.73% to 71.49%), surpassing HHD and VHHD countries with greater economic development. The exception was in Tropical Medicine, where authors from VHHD countries signed 75.24% of their papers in collaboration with researchers from other countries, compared to 58.34% in the case of MHD countries.

**Table 3:** Distribution of scientific contributions and the degree of international collaboration according to HDI of participating countries in documents included in the SCI-Expanded database in the categories of Tropical Medicine, Infectious Diseases, Parasitology and Pediatrics (2011–2015).

Human Development Index	Tropical Medicine		Infectious Diseases		Parasitology		Pediatrics	
	N docs	IC (%)	N docs	IC (%	N docs	IC (%)	N docs	IC (%)
Very high	9117	6860 (75.24)	54,144	20,803 (38.42)	20,158	11,053 (54.83)	63,309	11,228 (17.73)
High	7332	2457 (33.51)	13,568	5880 (43.34)	10,050	3975 (39.55)	11,058	2303 (20.83)
Medium	3394	1980 (58.34)	6851	4898 (71.49)	3694	2305 (62.4)	4728	1264 (26.73)
Low	3344	2853 (85.32)	5638	5007 (88.81)	3049	2636 (86.45)	1095	771 (70.41)

N docs: Number of documents; IC: International collaboration.

The analysis of international collaboration by geographic area and country (Table 2) confirmed our initial observations, with Africa (the region comprising the highest number of LHD and MHD countries) presenting the highest degree of international collaboration (between 50.52% of the documents in Pediatrics and 85.64% in Infectious Diseases). Europe, where all countries except Moldova present a high or very high HDI, showed a high degree of collaboration in Tropical Medicine (84.31%) and Parasitology (66.97%). The high collaboration indexes in Oceania (40.33% to 81.46%) and Latin America and the Caribbean (62.59% in Tropical Medicine and 50.6% in Infectious Diseases) are also notable. Moreover, we observed no correlation between the degree of overall collaboration in some geographic regions and the collaboration observed in their corresponding countries of reference (i.e. the biggest and most densely populated countries in the region). This is the case for Brazil in Latin America and the Caribbean and for China and India in Asia. These countries present considerably lower rates of international collaboration than their respective regions as a whole. In contrast, the three countries of the largest size and scientific development in Europe (the UK, Germany, and France), along with the United States in North America, present collaboration rates above their regions’ averages.

**Fig 3:**
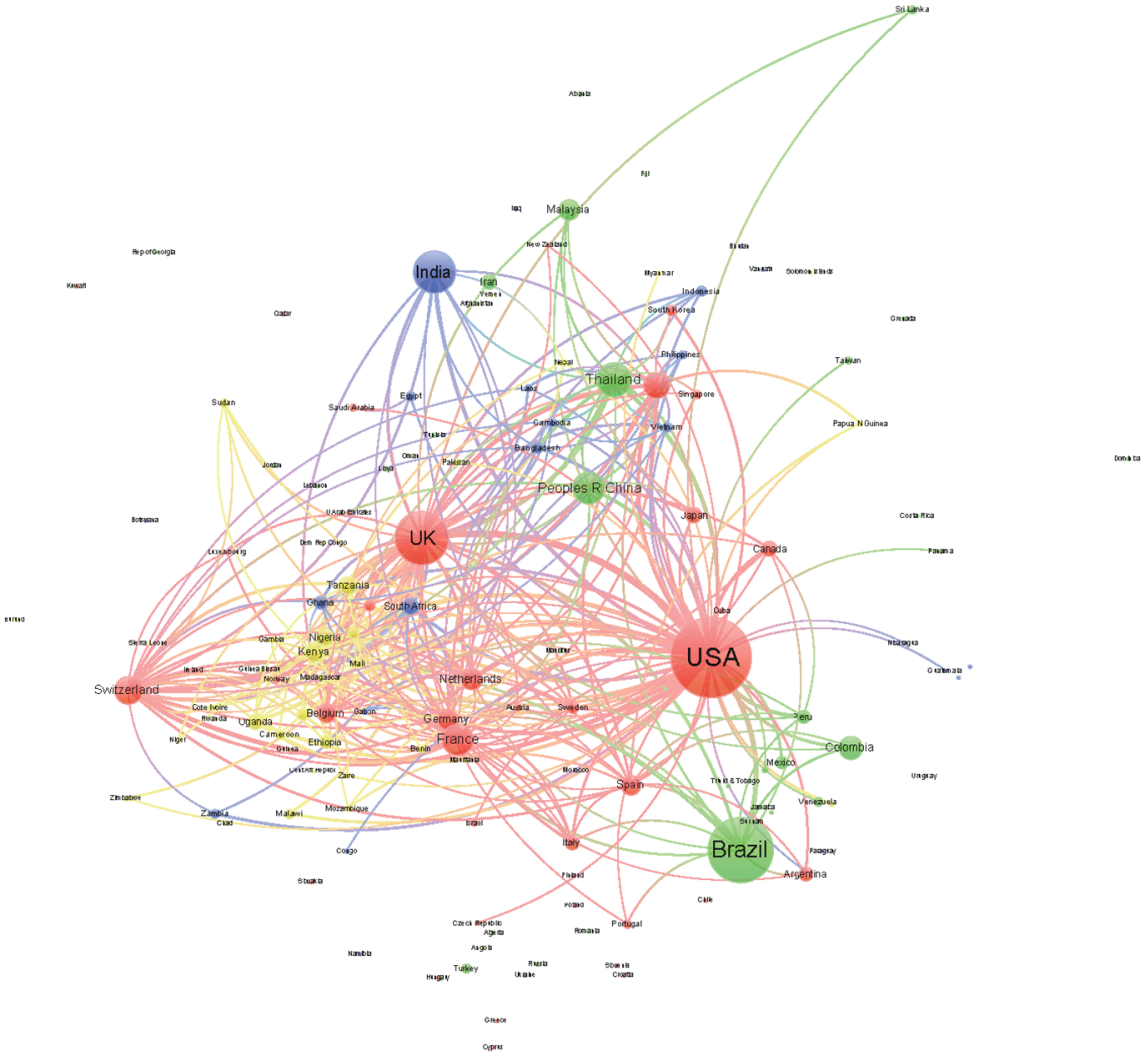
Network generated from international collaborations identified in documents included in the SCI-Expanded database in Tropical Medicine (2011–2015). Colors represent HDI of the countries (red: VHHD; green: HHD; blue: MHD; and yellow: LHD).

**Fig 4:**
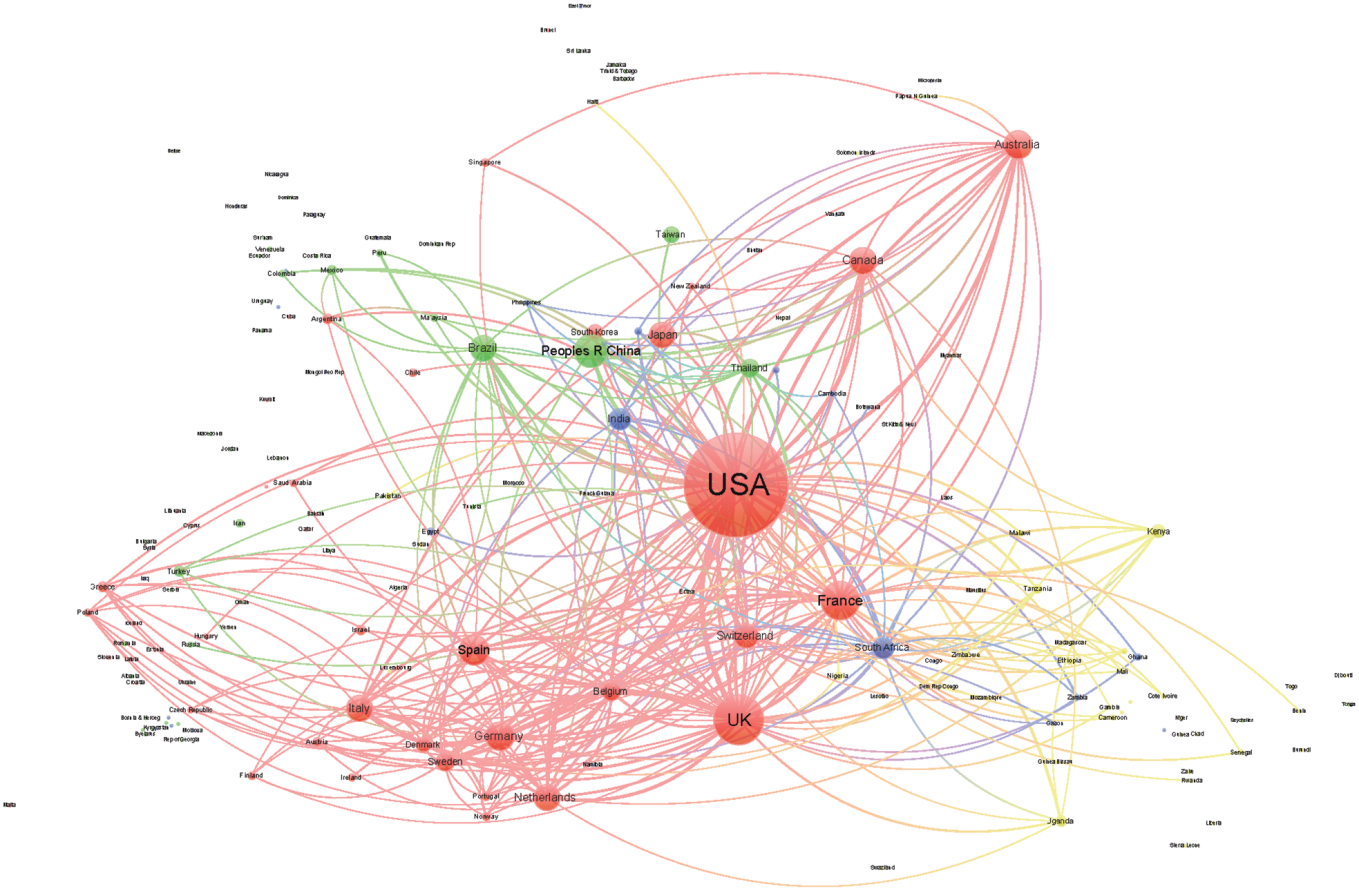
Network generated from international collaborations identified in documents included in the SCI-Expanded database in Infectious Diseases (2011–2015). Colors represent HDI of the countries (red: VHHD; green: HHD; blue: MHD; and yellow: LHD).

**Fig 5:**
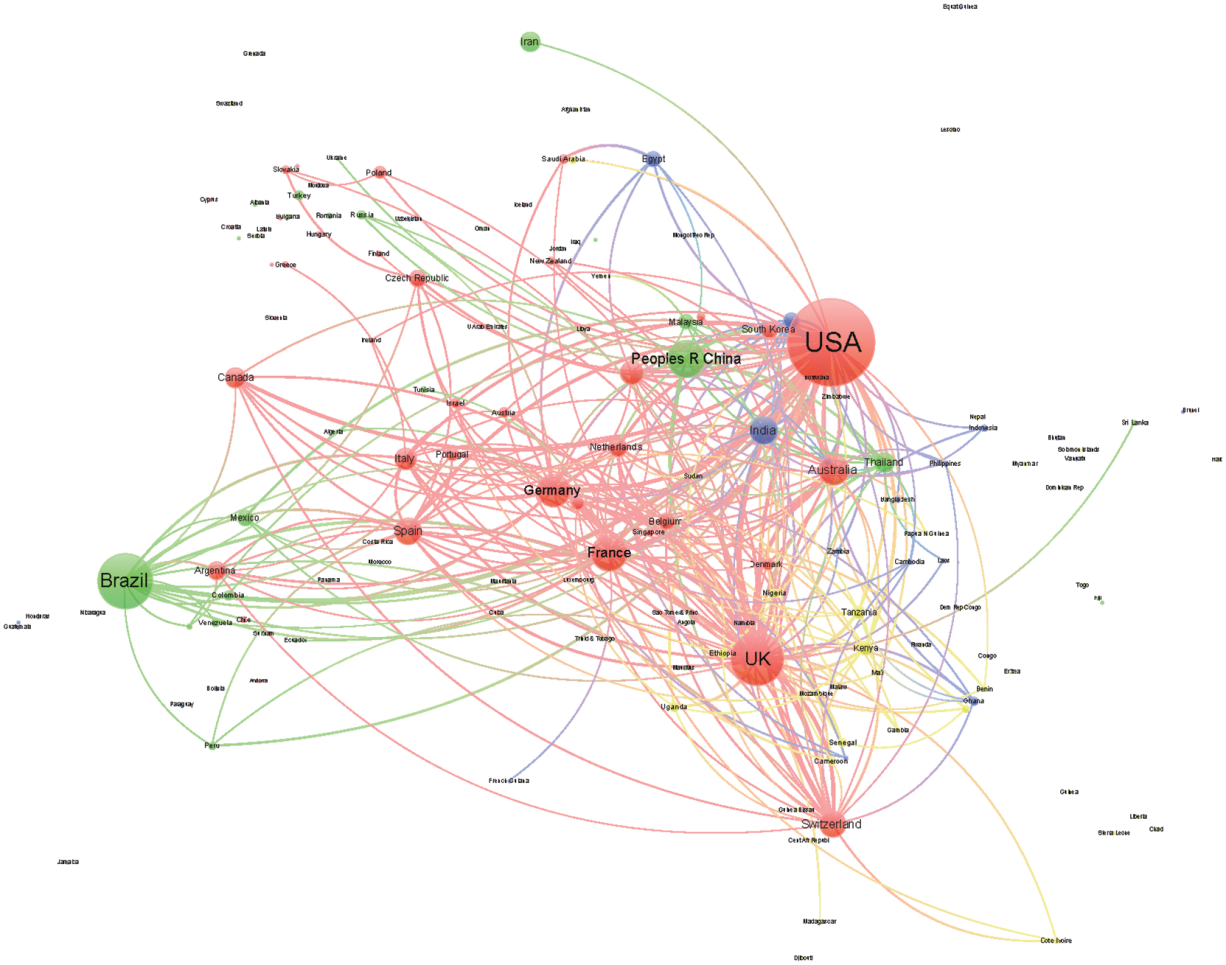
Networks generated from international collaborations identified in documents included in the SCI-Expanded database in Parasitology (2011–2015). Colors represent HDI of the countries (red: VHHD; green: HHD; blue: MHD; and yellow: LHD).

**Fig 6:**
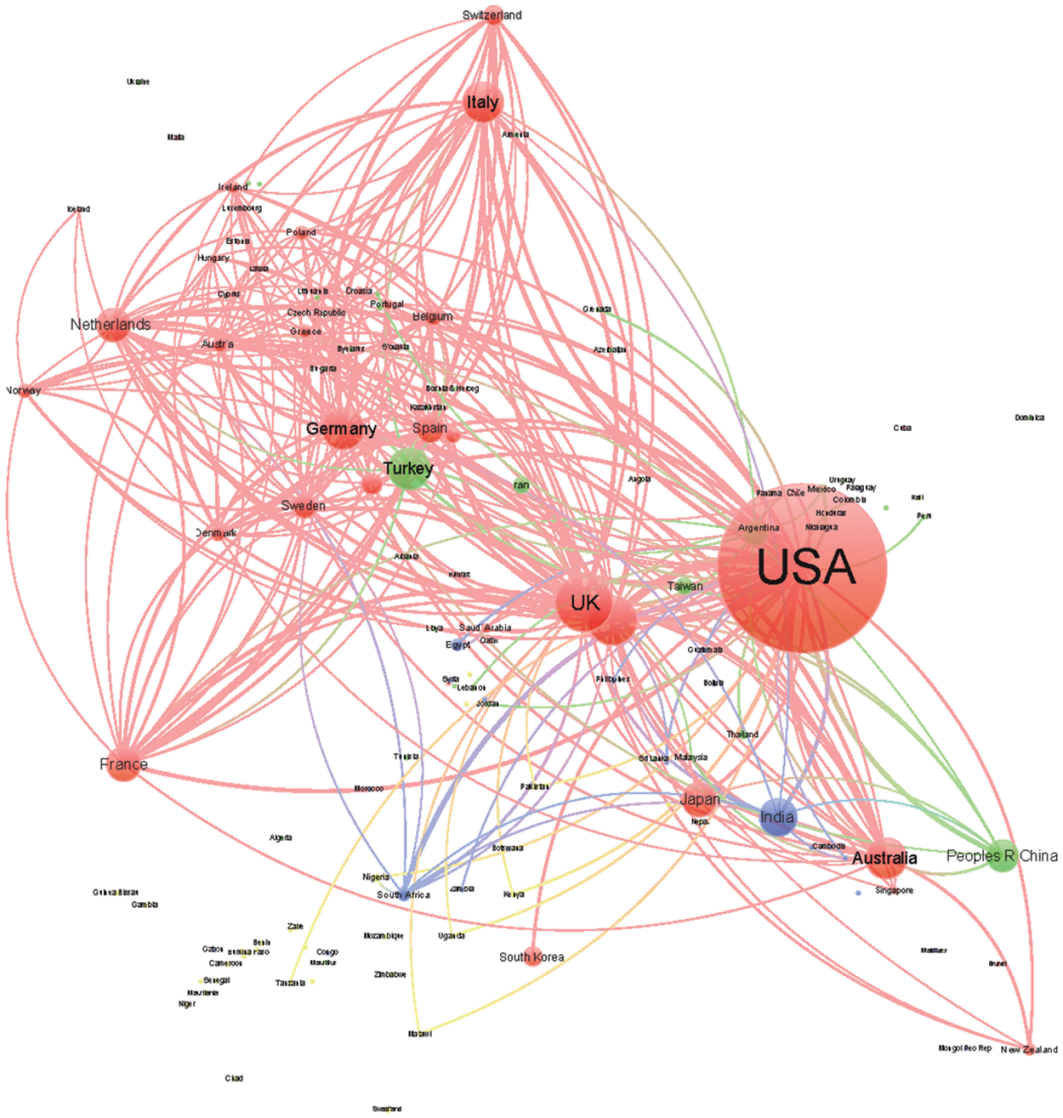
Networks generated from international collaborations identified in documents included in the SCI-Expanded database in Pediatrics (2011–2015). Colors represent HDI of the countries (red: VHHD; green: HHD; blue: MHD; and yellow: LHD).

Figs 3–6 shows the international collaboration networks of the countries in the four research areas analyzed. In general, the VHHD countries (the United States, Canada, Australia, the UK, and other European countries) occupy central positions within all of the networks, as authors from these countries have established the largest number of cooperative links and present the most intense collaborations with authors from other countries. Other countries rarely stand out. In Tropical Medicine and to a lesser extent in Infectious Diseases and Parasitology, some HHD countries like Brazil, China, and Thailand occupy a relatively prominent position, mainly as a result of the collaborations that their researchers have established with authors in the United States. The same is true for some MHD countries (India, South Africa, and Ghana). LHD countries (Tanzania, Kenia, Nigeria, Uganda, and Ethiopia) only occupy prominent positions within the network in the area of Tropical Medicine, in these cases linked to collaborations with different European countries. In the area of Pediatrics, the hegemony of the United States is unmatched, whereas MHD and LHD countries have scant representation.

In all of the research areas analyzed, the United States and the UK show the highest degree of betweenness, followed by European and other VHHD countries (Table 4). The analysis of the distribution of collaborations according to the HDI of the countries with which the collaboration took place shows asymmetrical distributions with different patterns of collaboration. For example, using Tropical Medicine as a reference, Brazil and China collaborate primarily with VHHD countries (74.9% to 75.2% of the documents with their participation) and occasionally with MHD and LHD countries (5.89% to 9.14%). On the other hand, the United States, the UK, and other VHHD countries like France present a much higher degree of collaboration in Tropical Medicine with LHD (24.55% to 29.27%) and MHD countries (11.13% to 17.65%). For their part, MHD and LHD countries present a moderate degree of collaboration with countries at similar levels of human development (14.2% to 25.99% of existing collaborations), although some countries like India, Nigeria and Pakistan principally collaborate with VHHD countries (in 55.88% to 67.17% of the papers published in Tropical Medicine). The same general patterns of collaboration hold for the rest of the categories analyzed, with the exception of the few links observed between VHHD countries and MHD/LHD countries (Table 4).

**Table 4:** Centrality of the countries analyzed in the international collaboration networks and distribution of their existing collaborations according to the HDI of the countries with which they collaborated, in documents included in the SCI-Expanded database in the categories of Tropical Medicine, Infectious Diseases, Parasitology and Pediatrics (2011–2015).

Country (HDI)	Cat	Betweenness (rank)	Number of different countries with which a collaboration has taken place (degree), number of collaborations (∑ N links), and % of total documents in collaboration, according to HDI groups											
			Low HDI			Medium HDI			High HDI			Very high HDI		
			Degree	∑ N links	% cols	Degree	∑ N links	% cols	Degree	∑ N links	% cols	Degree	∑ N links	% cols
Australia (VHHD)	TM	0.017525 (14)	30	235	16.29	16	237	16.42	16	250	17.32	25	721	49.96
	ID	0.016153 (10)	36	425	8.6	22	615	12.45	27	749	15.16	40	3151	63.78
	PA	0.015473 (14)	20	225	11.18	17	235	11.68	22	333	16.55	30	1219	60.59
	PE	0.029586 (8)	15	47	1.98	15	136	5.72	18	228	9.59	38	1966	82.71
Brazil (HHD)	TM	0.024377 (9)	23	68	5.89	16	70	6.06	17	148	12.82	28	868	75.22
	ID	0.010280 (16)	27	190	6.35	24	283	9.46	30	503	16.81	43	2016	67.38
	PA	0.034273 (6)	22	66	3.53	19	94	5.03	24	244	13.07	38	1463	78.36
	PE	0.008905 (20)	5	9	1.1	12	47	5.73	25	92	11.22	38	672	81.95
France (VHHD)	TM	0.050658 (3)	34	518	29.27	16	197	11.13	28	229	12.94	33	826	46.67
	ID	0.046113 (2)	40	1060	13.63	28	668	8.59	42	802	10.31	43	5249	67.48
	PA	0.053199 (4)	33	544	17.87	20	290	9.53	33	427	14.03	39	1783	58.57
	PE	0.049096 (3)	22	58	2.32	12	60	2.4	28	176	7.05	40	2203	88.22
Germany (VHHD)	TM	0.033178 (5)	29	191	18.12	17	186	17.65	22	121	11.48	32	556	52.75
	ID	0.018820 (7)	33	378	6.17	29	437	7.13	35	492	8.03	43	4821	78.67
	PA	0.054721 (3)	33	185	7.26	23	303	11.9	29	292	11.46	41	1767	69.37
	PE	0.030909 (7)	13	23	0.65	17	81	2.3	32	240	6.83	43	3169	90.21
India (MHD)	TM	0.023478 (10)	25	122	14.2	11	69	8.03	15	91	10.59	30	577	67.17
	ID	0.008947 (20)	29	323	12.07	23	320	11.96	32	431	16.11	38	1602	59.86
	PA	0.005116 (30))	22	92	11.25	12	66	8.07	17	78	9.53	29	582	71.15
	PE	0.029222 (9)	14	63	7.67	17	72	8.77	25	93	11.33	35	593	72.23
Japan (VHHD)	TM	0.010172 (19)	21	52	10.12	17	97	18.87	14	167	32.49	22	198	38.52
	ID	0.005064 (30)	25	93	5.93	20	295	18.81	24	400	25.51	40	780	49.74
	PA	0.022381 (9)	21	90	8.1	21	219	19.71	19	291	26.19	29	511	45.99
	PE	0.006834 (25)	6	11	1.79	11	47	7.65	19	86	14.01	34	470	76.55
Nigeria (LHD)	TM	0.007591 (25)	27	112	25.99	13	41	9.51	11	26	6.03	22	252	58.47
	ID	0.004606 (35)	32	199	23.44	16	111	13.07	13	62	7.3	33	477	56.18
	PA	0.006211 (27)	19	77	22.65	15	34	10	9	28	8.24	22	201	59.12
	PE	0.005804 (26)	12	27	13.85	8	45	23.08	13	24	12.31	21	99	50.77
Pakistan (LHD)	TM	0.002220 (44)	11	16	15.69	7	16	15.69	6	13	12.74	18	57	55.88
	ID	0.004168 (38)	21	70	10.36	19	116	17.16	25	132	19.53	39	358	52.96
	PA	0.001576 (59)	7	8	6.35	4	8	6.35	9	19	15.1	23	91	72.22
	PE	0.004046 (34)	8	19	8.41	12	51	22.57	10	21	9.29	21	135	59.73
China (HHD)	TM	0.007613 (24)	17	32	6.09	14	48	9.14	13	52	9.9	26	393	74.86
	ID	0.005771 (27)	23	116	4.16	22	262	9.39	28	352	12.61	41	2061	73.84
	PA	0.010664 (19)	20	56	4.45	15	87	6.92	17	109	8.67	33	1005	79.95
	PE	0.006932 (24)	6	19	2.32	9	47	5.73	18	57	6.95	34	697	85
South Africa (MHD)	TM	0.011215 (17)	28	152	24.01	14	61	9.64	12	26	4.11	26	394	62.24
	ID	0.012876 (14)	36	764	18.27	23	310	7.41	28	272	6.5	40	2835	67.81
	PA	0.022368 (10)	24	160	16.67	14	65	6.77	21	74	7.71	33	661	68.85
	PE	0.025028 (11)	16	89	12.19	11	59	8.08	19	57	7.81	38	525	71.92
UK (VHHD)	TM	0.094007 (2)	37	1194	26.17	23	662	14.51	29	592	12.97	35	2115	46.35
	ID	0.041683 (3)	41	1827	14.15	32	1402	10.86	42	1227	9.5	44	8453	65.48
	PA	0.100268 (2)	39	1138	19.39	25	612	10.43	36	784	13.36	43	3334	56.82
	PE	0.069292 (2)	24	223	4.37	17	246	4.82	39	377	7.39	44	4254	83.41
USA (VHHD)	TM	0.117987 (1)	37	1394	24.55	24	883	15.55	34	1191	20.97	33	2211	38.93
	ID	0.083418 (1)	42	2977	15.45	35	2771	14.38	47	3485	18.09	43	10036	52.08
	PA	0.135182 (1)	40	1059	13.82	30	791	10.32	40	1638	21.37	41	4175	54.48
	PE	0.187711 (1)	33	424	5.1	24	671	8.07	39	1310	15.75	44	5910	71.07

Cat: category; TM: Tropical Medicine; ID: Infectious Diseases; PA: Parasitology; PE: Pediatrics; % cols: % of all collaborations.

The analysis of influence in the cooperative networks, presented through the matrix of directed links and the calculation of the dominance indexes (Table 5), sheds light on some aspects of interest in the characterization of cooperative practices. In that sense, the prominent influence or dominance exercised by Brazil and China in their cooperative links with the United States, the UK and other European countries is notable. This pattern of Brazilian and Chinese dominance does not hold for their collaborations with other countries in the geographic vicinity, perhaps due to the scant cooperative links that exist (for example, Chinese authors have only participated in three documents in collaboration with authors from India and in one with authors from Pakistan) or because the collaborative relationships are more balanced (for instance, Brazilian researchers have led 20 papers with participation from Colombian authors and participated in 16 papers led by Colombians). On the other hand, the United States maintains its influence or dominance in collaborations with different VHHD countries such as Australia, France, or Japan, as well as with MHD or LHD countries like Nigeria. Other countries, including India, South Africa and Pakistan, maintain a more balanced collaborative relationship with the United States.

**Table 5:** Matrix with collaboration ties and dominance indexes in Tropical Medicine publications, in documents included in the SCI-Expanded database (2011–2015).

Dominance Indexes N collaborations	Australia	Brazil	France	Germany	India	Japan	Nigeria	Pakistan	China	South Africa	UK	USA
Australia		↑ 0.9	↑ 0.67	↑ 0.57	↑ 0.7	← 1	= 0.5	↑ 1	↑ 0.6	↑ 0.62	← 0.53	↑ 0.58
Brazil	↗18 ↙2		← 0.68	← 0.61	↑ 1	← 1	↑ 0.75	↑ 1	= 0.5	-	← 0.78	← 0.74
France	↗12 ↙6	↗10 ↙21		↑ 0.71	↑ 0.58	← 0.57	↑ 1	↑ 1	↑ 0.8	← 0.83	← 0.56	↑ 0.67
Germany	↗4 ↙3	↗9 ↙14	↗15 ↙6		← 0.54	= 0.5	← 0.64	← 0.67	← 0.75	← 0.7	← 0.69	= 0.5
India	↗12 ↙5	↗1 ↙0	↗7 ↙5	↗6 ↙7		↑ 1	↑ 1	—	← 0.67	← 1	← 0.51	↑ 0.51
Japan	↗0 ↙1	↗0 ↙7	↗3 ↙4	↗1 ↙1	↗2 ↙0		↑ 0.67	↑ 1	← 0.6	—	← 0.78	↑ 0.62
Nigeria	↗2 ↙2	↗3 ↙1	↗2 ↙0	↗4 ↙7	↗2 ↙0	↗2 ↙1		—	↑ 1	← 1	←0.58	↑ 0.65
Pakistan	↗1 ↙0	↗1 ↙0	↗2 ↙0	↗1 ↙2	↗0 ↙0	↗1 ↙0	↗0 ↙0		← 1	← 1	← 0.58	↑ 0.53
China	↗23 ↙15	↗1 ↙1	↗12 ↙3	↗1 ↙3	↗1 ↙2	↗7 ↙9	↗1 ↙0	↗0 ↙1		—	← 0.69	← 0.67
South Africa	↗5 ↙3	↗0 ↙0	↗1 ↙5	↗3 ↙7	↗0 ↙3	↗0 ↙0	↗0 ↙1	↗0 ↙1	↗0 ↙0		↑ 0.7	↑ 0.51
UK	↗43 ↙48	↗19 ↙67	↗34 ↙44	↗16 ↙35	↗25 ↙26	↗2 ↙7	↗5 ↙7	↗5 ↙7	↗11 ↙25	↗46 ↙20		↑ 0.52
USA	↗62 ↙44	↗71 ↙200	↗50 ↙24	↗20 ↙20	↗65 ↙63	↗18 ↙11	↗39 ↙21	↗9 ↙8	↗36 ↙73	↗29 ↙28	↗199 ↙186	

N Collaborations: ↗ Number of first authorships in collaborative documents; ↙ Number of collaborative documents without participation as lead author. Dominance indexes: ↑ Dominance index in favor of country listed in top row; ← Dominance index in favor of country included in lefthand column; = Authors from both countries have signed the same number of documents in the first position;—: no collaborative links on papers led by authors from one of the two countries.

Outside the United States, relationships between VHHD countries are irregular, with no clear pattern of leadership or dominance. For example, Germany appears dominant over other European countries (France and the UK) in their joint research work, but its ties with the United States are more equitable. Likewise, the UK maintains equitable relations with the United States and Australia but is dominated by other European countries such as France and Germany. One final aspect to highlight is the scant collaborations existing between countries from different regions, beyond the collaborations that exist among VHHD countries, described above.

**Fig 7:**
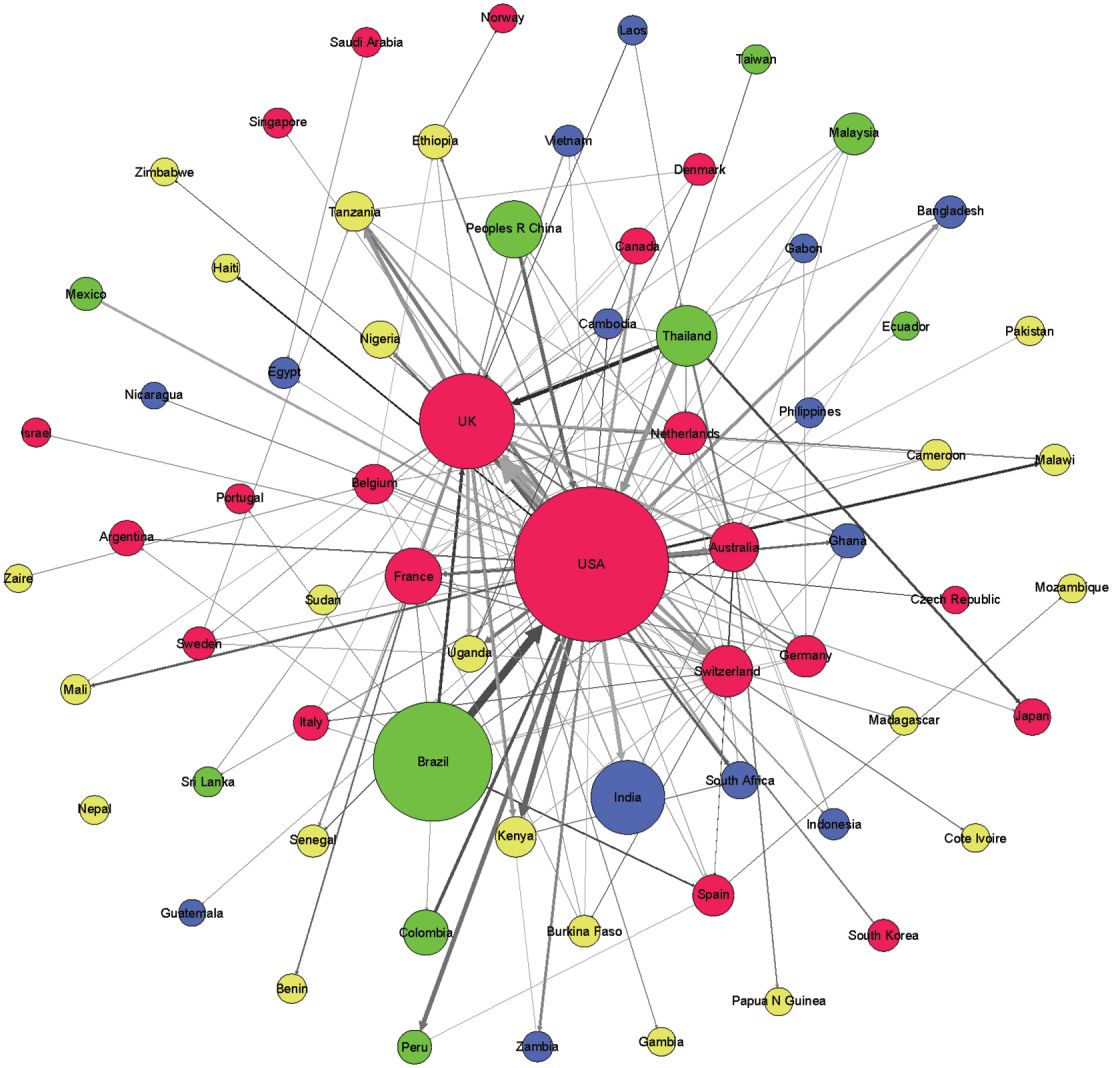
Directed network generated based on the dominance indexes in the Tropical Medicine research area, in documents included in the SCI-Expanded database (2011–2015). Colors represent HDI of the countries (red: VHHD; green: HHD; blue: MHD; and yellow: LHD).

The directed network constructed based on the dominance indexes (Fig 7) enables a deeper look into other aspects of interest. For example, in papers led by some HHD countries, the participation of authors from the United States and the UK serves to favor the hegemony and dominance of the latter two within the collaboration networks. We have already mentioned Brazil and China in this regard, but the same is true for collaborations with other countries, such as Malaysia, Thailand, Colombia, and Mexico. It is also worth noting that the United States has a subordinate role in relation to some VHHD countries in Asia and Latin America that are smaller in size and population density but have advanced scientific systems; examples include South Korea and Israel. Finally, we highlight the fact that collaborating with VHHD countries that exert dominance or leadership—namely the United States and the UK, but also other European countries, Canada, and Australia—is essential in ensuring the participation and integration of less developed countries in research networks, as these present a low degree of collaboration with other countries.

### Contribution and leadership in research activities

The analysis of the leadership exercised by country groups based on HDI in terms of their relative contributions to the overall number of signatures and their presence as first and/or corresponding authors (S6 Table), illustrates the predominance of VHHD countries, which are responsible for 46.27% of the signatures in the case of Tropical Medicine and 81.25% in Pediatrics. These values contrast with the limited weight of MHD and LHD countries, which is especially significant in the case of Pediatrics, as these countries only contribute 5.25% and 1.31% of the total signatures, respectively. Indeed, only in Tropical Medicine do MHD and LHD countries participate more intensely, together contributing 26.86% to the total signatures, but in any case, this value is far from proportional given their share of the world population (46%). This gap widens when assessing their presence in the first author position and the address for correspondence. In contrast, HHD countries contribute 38.92% of the lead authorships, despite a more modest share of the total signatures (26.85%). In the rest of the categories, these differences are even more pronounced (S6 Table).

The analysis of leadership in the countries, considering the documents in which they are represented and their HDI, shows that authors from LHD countries lead 40.55% to 55.89% of the total papers they sign, depending on the research area. In contrast, authors from VHHD countries lead 68.5% to 97.01% of their total contributions. This pattern of distribution repeats with regard to participation as corresponding authors. Another salient point emerging is the elevated degree of leadership exercised by HHD countries, which rank highly in both of these parameters (Table 6).

**Table 6:** Participation as first author and corresponding author, by HDI in country of origin, in documents included in the SCI-Expanded database in the categories of Tropical Medicine, Infectious Diseases, Parasitology and Pediatrics (2011–2015).

Research Area		Very High HDI	High HDI	Medium HDI	Low HDI
		% docs	% docs	% docs	% docs
Tropical Medicine	1^st^ position	68.5	86.21	63.82	45.10
	Corresponding author	71.54	85.45	60.99	40.46
Infectious Diseases	1^st^ position	89.17	78.32	53.53	40.55
	Corresponding author	90.31	76.7	50.49	36.18
Parasitology	1^st^ position	82.16	82.88	58.04	46.41
	Corresponding author	83.56	82.96	54.98	43.16
Pediatrics	1^st^ position	97.01	90.01	84.58	55.89
	Corresponding author	97.39	88.71	83.08	51.23

Docs: documents.

**Fig 8:**
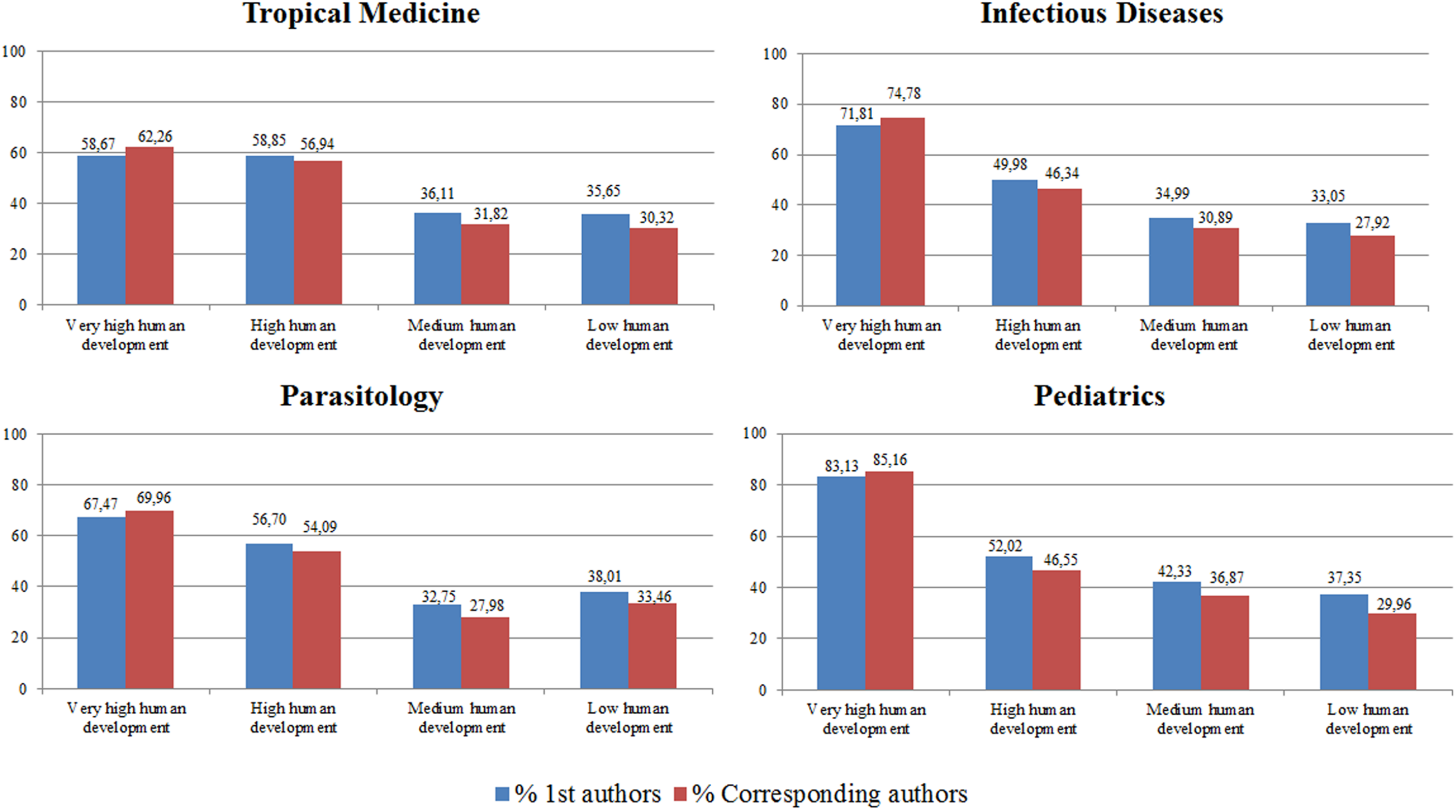
Participation as first author and corresponding author (% of documents) in papers produced in international collaboration, by HDI in country of origin, in documents included in SCI-Expanded Database in the categories of Tropical Medicine, Infectious Diseases, Parasitology and Pediatrics (2011–2015).

This same analysis of leadership, considering only the documents produced with international collaboration (Fig 8), shows that LHD countries have an even lower weight with regard to their authors’ presence in the position of first author (33.05% to 38.01% of the collaborative documents), and this drops further still when examining their participation as corresponding authors (27.92% to 33.46%). The values for MHD countries are similar, reflecting limited leadership in collaborative papers (32.75% to 42.73% of first authorships and 27.98% to 36.87% of corresponding authorships). In addition to the clear leadership of VHHD countries, it is interesting to note that these countries are more frequently represented in the address for correspondence than in the position of first author.

### Impact of papers according to leadership and type of collaboration

The analysis of the citation degree by collaboration type (Table 7) shows that the most cited papers are led by VHHD/HHD countries with the simultaneous participation of another VHHD/HHD country and MHD/LHD countries (type 7). Although the papers on which only MHD/LHD countries participate (types 3 and 4) present the lowest citation degrees, the opposite is the case for those led by MHD/LHD countries with the participation of both MHD/LHD and VHHD/HHD countries (type 8)—indeed, their citation degrees are just below the levels seen for the papers produced only by authors from VHHD/HHD countries (type 2), and in the case of Tropical Medicine, they are even higher. Papers led by MHD/LHD countries with participation from VHHD/HHD countries (type 6) also show high degrees of citation.

**Table 7:** Average citations per paper group by collaboration types, in documents included in SCI-Expanded database in the categories of Tropical Medicine, Infectious Diseases, Parasitology and Pediatrics (2011–2015).

Collaboration types			WoS Subject category			
Type	First position	Second and subsequent positions	Tropical Medicine mean ± SD	Infectious Diseases mean ± SD	Parasitology mean ± SD	Pediatrics mean ± SD
1	VHHD/HHD	-	3.49 ± 6.59	6.69 ± 23	6.81 ± 10.76	3.37 ± 6.53
2	VHHD/HHD	VHHD/HHD	4.22 ± 6.97***	6.98 ± 13.59†	6.7 ± 11.57†	4.16 ± 7.93***
3	MHD/LHD	-	2.4 ± 4.57***	3.18 ± 5.17***	3.77 ± 6.24***	1.83 ± 3.35***
4	MHD/LHD	MHD/LHD	2.65 ± 4.51***	3.46 ± 5.43***	3.78 ± 5.34***	1.89 ± 3.48***
5	VHHD/HHD	MHD/LHD	5.52 ± 9.93***	7.31 ± 11.99†	5.81 ± 9.89***	3.71 ± 5.06†
6	MHD/LHD	VHHD/HHD	4.79 ± 8.7***	6.25 ± 10.14†	5.72 ± 9.66*	3.0 4 ± 4.45†
7	VHHD/HHD	VHHD/HHD + MHD/LHD	6.56 ± 10.28***	8.37 ± 14***	7.16 ± 11.3†	4.76 ± 7.7***
8	MHD/LHD	MHD/LHD + VHHD/HHD	5.28 ± 7.48***	6.35 ± 10.14^†^	5.82 ± 10.64**	3.51 ± 5.18^†^

SD: Standard deviation; We considered reference citation values as mean degree of citation obtained by VHHD/HHD countries (type 1), comparing these in descending order with means obtained by other collaboration types in each research area.

Significance:

^†^ not significant

* p <0.05

** p<0.01

***p<0.001

Both the statistical analyses comparing the mean citation degree between collaboration type 1 versus types 2 to 8 (Table 7) as well as the analysis of variance performed to compare each pair of collaboration types against each other (S7 Table) show statistically significant differences between most categories. Specifically, the mean citation degrees of papers produced solely by MHD/LHD countries are well below those of papers produced solely by VHHD/HHD countries (collaboration types 3 and 4 versus types 1 and 2). However, it is worth highlighting that the papers led by MHD/LHD countries with participation from VHHD/HHD countries (type 6) do not present significant differences with the papers led by VHHD/HHD with participation from MHD/LHD countries (type 5) in any of the areas analyzed. On the other hand, the papers led by VHHD/HHD countries with participation from both other VHHD/HHD and MHD/LHD countries (type 7) do show a significantly higher mean citation degree compared to those led by MHD/LHD countries with participation from both other MHD/LHD countries and VHHD/HHD countries (type 8) for all research areas (*p* < 0.001). This is particularly notable given the high mean citation degree obtained for type 8 publications relative to every other type except type 7.

## Discussion

### The order of signatures and the address for correspondence as a measure of dominance and leadership in research activities

A diverse body of literature exists that has quantified the research contributions of less developed countries and regions through authorship in scientific publications, analyzed their collaboration and integration in the international collaboration networks, and studied the citation degree of documents in the research areas covered here [29,35–41]. However, these analyses do not consider or integrate any data on the order of the authors’ signatures or the address for correspondence. Our study shows that these bibliographic characteristics may provide highly relevant information that can enrich any analysis of the role played by different participants in scientific publications and of the interactions established in collaborative research work.

In addition, the studies of international scientific collaboration based on network analysis generally assume that collaboration is a balanced, bi-directional process for interaction. These studies analyze the role played by countries within the networks by calculating indicators that consider the total number of established collaborations or the number of countries represented among the collaborators. But the collaborations between two given countries may not respond to a model of equitable cooperation; on the contrary, the dominant feature of their collaboration may be imbalance or diversity. This description is evident in some of the cooperative interactions analyzed in the present study, which considers the number of documents signed by first authors from each pair of collaborating countries. To characterize this diversity in our analysis of scientific collaborations, we have invoked the concept of *dominance* used in areas such as Ecology or Economics to describe the degree of equilibrium or proportion in the interaction between the objects of analysis. Other authors refer to this notion of dominance as “balance”, “evenness”, “equitability” or “importance” [42,43]. Capturing this concept through indicators such as the dominance index, introduced in the present study, enables a far more precise analysis and interpretation of the collaborative links identified through scientific publications.

### Contributions to research and scientific collaboration networks according to the HDI of participating countries

Although different papers have reported increased participation from less developed countries in scientific publications in the areas of Tropical Medicine, Infectious Diseases and Parasitology [44–46], our study reveals that a high degree of scientific dependence and subordination persists, with these countries exercising limited leadership in research activities in the areas studied. Keiser and Utzinger [29] analyzed five high-impact journals on Tropical Medicine from 1952 to 2002, reporting a persistently low presence of authors from LHD countries (11.4% in 1952 and 13.4% in 2002). Similarly, Keiser et al. [4] analyzed six top journals on Tropical Medicine from 2000 to 2002, finding that only 12.5% of the documents had a first author from a low-income country (a measure that is roughly equivalent to LHD countries but based on countruies’ gross national income) [47]. This is generally consistent with the 9.3% reported in our study in that research area, suggesting that there has in fact been a decline in LDH authors’ leadership in recent years. Other studies have also reported the scant representation of these authors in other biomedical research areas. Adam et al. [28] found that in scientific production on health policy and systems research, just 4% of the papers published between 2003 and 2009 were led by authors from low-income countries, while Wong et al. [48] reported that only 17% of the papers describing randomized controlled trials in oncology between 1998 and 2008 had first or corresponding authors from low- and middle-income countries.

To explain the limited participation of low-income countries that endures in research activities despite the increases observed in some studies, authors tend to cite the center-periphery model of research development, recognizing the existence of considerable socioeconomic inequalities [21]. Health research spending is undeniably disproportionate, neglecting the poorest populations and their health concerns. In 1990, the Global Forum for Health Research coined the term “10/90 gap” to describe a situation in which less than 10% of worldwide resources devoted to health research were put towards health in developing countries, where over 90% of all preventable deaths worldwide occurred. This spending gap is a decisive factor affecting the potential of less developed countries to carry out research activities, despite the numerous initiatives that aim to promote investments to that end [1,49].

More specifically, Smith et al. [50] propose different factors that may help to explain the limited contributions and leadership among authors from low-income countries. One is the greater share of responsibility that these authors have for technical tasks related to the work performed in the collaboration; these activities receive less recognition in terms of authorship and have less to do with drafting the research reports. Indeed, manuscript preparation is a key determinant of the right to authorship and the order of the same, but many of the researchers in low-income countries have a low level of English. It is also possible that the power dynamics between researchers from high-income countries and low- and middle-income countries allow space for unfair practices or publication biases that favor papers from prominent Western researchers in the peer-review evaluations [50–52]. It is also possible that the increase in scientific production observed in all geographic regions in the areas of Tropical Medicine, Infectious Diseases, and Parasitology, including those with a larger presence of low- and middle-income countries [29,35,36], could be the result of multinational or multiregional collaborations, masking a phenomenon of subordination and limited leadership in researchers from less developed countries [53]. Thus, it is important to develop precise indicators that measure the research contributions of authors from different countries and geographic regions as well as their participation in international collaboration networks.

The networks generated in this study, showing collaboration links between different countries, illustrates the continuity of some of the characteristic features of collaboration that previous studies have described. In that sense, it is worth highlighting the centrality of the core countries with more scientific development, particularly the United States and the UK along with Canada, Australia, Japan, and other VHHD countries in Europe. This preeminent position has been reinforced by the increased links among this group of countries as well as by their ability to access, absorb, and make use of participants from peripheral countries [54,55]. Another specific aspect of the collaboration between VHHD countries is the high degree of collaboration and the density of links displayed between different European countries. This can be interpreted as the result of the European Commission initiatives catalyzing cooperative practices, for example through the European Framework Programmes and through programs to promote researcher mobility within the European Union [56]. Other factors such as geographic proximity are also at play.

In the specific area of Tropical Medicine as well as in Parasitology, the high level of collaboration between European countries can be attributed to the traditional interest of these countries in these diseases and their social and linguistic connections to former colonies in less developed regions of the world [36]. Moreover, international collaboration continues to be the mechanism through which less developed countries are integrated into research activities, as reflected in the high rates of international collaboration pertaining to MHD and LHD countries in our study. However, this phenomenon is more frequent in smaller countries, which tend to have more limited scientific systems; in contrast, Nigeria and India show collaboration rates that are considerably lower than their regional averages. Other studies have suggested that the main factor explaining this pattern is the greater difficulty in establishing collaborations in small communities, increasing these researchers’ dependence on international networks [57–59].

The present study has identified some novel aspects of cooperative practices, with the cases of Brazil and China emerging as especially relevant. The international collaboration of Brazil shows some factors that are very different from those reported by Glänzel et al. [60] for the 1991–2003 period. At that time, the level of collaboration that Brazil maintained with the United States and member states of the European Union was comparable with that between Brazil and other surrounding countries in Latin America and the Caribbean. However, in our period of study (2011–2015), Brazil showed a pronounced orientation toward international collaboration with prominent research actors and much more modest involvement in research carried out in conjunction with its neighbors. For example, we found that researchers in Brazil co-signed 37.69% of their collaborative papers in Tropical Medicine with authors in European countries and 28.34% with authors in North America, compared to just 16.46% of the papers with researchers from other countries in Latin America and the Caribbean. On the other hand, the second-most productive country in this geographic region (Colombia) collaborated with neighboring countries in 35.01% of its papers, with North America in 29.18%, and with Europe in 20.42%. This situation responds to a lack of stable, well-supported programs for scientific collaboration within Latin America and to a strategy based on strengthening ties to scientifically central countries, particularly the United States [61]. Another novel aspect of interest is the fact that Brazil was the dominant partner in the collaboration links established with the United States and its second collaborator in volume of papers after the UK. In the rest of the research areas analyzed, we observed a similar situation albeit with diverse levels of dominance.

China exhibits the same features in international collaboration as Brazil, having established 31.24% of its collaborations with European countries and 27.43% with North America, compared to only 22.28% with other Asian countries. Moreover, China emerges as the dominant partner and a prominent collaborator in the cooperative links established with the United States. China’s development since adopting its opening-up policy in 1978 has been spectacular in terms of the growth and visibility gained in the world’s scientific literature. Since the mid-2000s, it has held the second position in terms of publication activity worldwide, trailing only the United States [62]; the country has also expanded and strengthened its international collaboration. International collaboration rates are still below those of European and North American countries, and this, together with the high values observed for China in terms of its representation among corresponding authors (around 90%), reflects the autonomous development of its scientific system [56]. Another notable element that has contributed to China’s international collaborations is the relevance of the so-called “Chinese immigrant scientists”, as nearly half (45%) of the researchers in English-speaking countries who collaborate with Chinese authors have Chinese lineage [63].

Countries like India and South Africa share more equitable collaboration links with the United States and Europe as well as with the other countries in their geographic vicinity. Conversely, Russia is the only country in the BRICS bloc to show limited participation in the research areas analyzed, which may be attributed to their reliance on their own scientific system, infrastructure, people, and skills, with subsequently less presence in international databases and fewer ties to international collaborators [64,65]. Beyond the case of Russia, the BRICS bloc has begun altering the traditional vision of international collaboration based only on the center-periphery model [55], which fails to present a satisfactory vision regarding the function of semi-peripheral countries [21]. These have become prominent collaborators of VHHD countries, in many cases dominating the cooperative links established between them and reducing the functional role that the core countries play for those on the periphery. Considering all of the above aspects, the hegemony of the United States and to a lesser extent of other VHHD countries in the measures of centrality can be attributed to the overall weight of the collaborations they maintain with a myriad of countries across the globe, which Brazil and China lack, concentrating their collaborations with VHHD countries and especially with the United States.

Many cooperative links—particularly between VHHD and MHD/LHD countries—continue to display patterns consistent with the center-periphery model, that is, dominated by economic factors and mutual interests that may be derived from collaborations. However, to explain other examples, it is necessary to draw on geographic, historical, cultural and linguistic influences, as Adams et al. [66] has reported in the case of Africa, where numerous South-South partnerships tied to the immediate surroundings are seen as equally positive and necessary as North-South collaborations [67].

### Importance of establishing criteria that regulate author order in scientific publications

Different studies based on questionnaires administered to the authors of scientific research papers have shown the differential treatment and recognition afforded to the first author position. Baerlocher et al. [13] reported that the levels of participation were highest for first authors, followed by last and then second authors. Middle authors had lower levels particularly in conception, drafts of the manuscript, supervision, and being a guarantor. Similarly, Zbar & Frank [68] confirmed that other scientists were at least seven times more likely to consider that the first author performed most of the work or had an instrumental role in the performance of the study and the writing of the manuscript. More recently, Conte et al. [69] highlighted the dramatic rise in author pairs claiming a joint role as first authors; whereas this phenomenon did not even exist in some biomedical and clinical journals in 1990, by 2012 over 30% of their papers featured co-first authorships. This fact illustrates the recognition that the research community grants to the first authors of scientific publications. Indeed, this author position raises their visibility both symbolically and literally, as for example some citation styles used in scientific journals feature the lead author’s name and omit the names of any collaborators from the main text. Likewise, first authorship leads to greater recognition during academic evaluation and promotion processes [12,70], making it a highly relevant consideration for researchers.

Despite the clear importance of author position, the 2016 update of the *Recommendations for the Conduct*, *Reporting*, *Editing*, *and Publication of Scholarly Work in Medical Journals* does not make any reference to how author order should be determined, and in fact it eliminates the guideline present in previous editions that the corresponding author should be prepared to explain the presence and order of authors [7]. The lack of any criteria regulating author order constitutes a potential source of conflict [12] and may result especially disadvantageous for the recognition of junior co-authors or those with a lower academic status [71]. In that sense, Smith et al. [50] suggests that this lack of clarity could result in under-recognition of researchers from low- and middle-income countries, a trend which may help to explain the lower weight of MHD/LHD countries in the present study.

### Citation and types of collaboration

The study of factors associated to the citation degree of scientific publications is a topic that has generated a large volume of literature [72]. The present study builds on this body of evidence, adding an empiric example based on the comparison of the citation degree of countries according to their HDI and the leadership exercised in publications to which they have contributed. The low citation degrees observed in publications produced solely be MHD/LHD countries is quite noteworthy and may be explained by different factors, including the lower visibility and recognition for contributions from these countries at an international level; the local-national problems that the papers address, which may be of less interest to central research countries that dominate publications in the leading journals; or the tendency for these authors to also cite papers from their immediate surroundings less frequently. For example, Meneghini [73] reported that Brazilian authors tend to cite prominent international authors to the detriment of their compatriots. Other variables may also limit the citation degree, including linguistic barriers and editorial strategies oriented to maintaining or increasing citation indicators [74–77].

Conversely, international collaboration is generally positively associated with citation degree, as various studies have pointed out [78–80], although the high degree of collaboration observed in countries with participation from solely VHHD authors confirms Persson’s [81] observation that collaboration is especially important for small countries where opportunities for national collaboration are limited. In comparison, about half of all highly cited papers in large countries such as the United States are authored domestically.

The present study has shown that the type of collaboration established between and among countries with different HDIs has a clear impact on the citation degree. Interestingly, the papers led by MHD/LHD authors with the participation of VHHD/HHD authors have similar or even higher citation degrees as other forms of collaboration, reflecting the importance of promoting these types of collaborations as a way to integrate MHD/LHD countries as equals in international research activity, both in terms of leadership and of impact. Other studies, including Confraria & Godinho [82] (2015) and Confraria et al. [83], have determined that concentrating available resources in a specialized field and creating long-lasting, international partnerships are other ways that MHD/LHD countries can increase the impact of their research output.

### Limitations, alternative approaches and future lines of research

Our study must assume the limitations inherent to all research based on the quantification of the bibliographic characteristics of scientific publications [84]. Moreover, there are other methodological approaches that measure the interactions between countries at different levels of economic and human development based on the study of co-authorships, for example, by assigning different weights to contributions based on the number of authors from each country that participate on the papers, or conferring more recognition on the corresponding authors, as these have a pivotal role in initiating and organizing the study [63]. It is also possible to analyze phenomena such as “neo-colonial science”, identifying the research that addresses specific problems associated with less developed countries but without the participation of authors from those countries [85].

### Conclusions

We observed little participation of authors from less developed (MHD/LHD) countries in mainstream journals covering Tropical Medicine, Infectious Diseases, Parasitology, and Pediatrics, even though these four research areas are acutely relevant to the health systems of these countries, which bring together nearly half of the global population. In this context, the participation of these countries in research activity should be promoted, particularly those initiatives that consider their interests and priorities [86,87]. The development of consolidated academic partnerships may be an excellent mechanism to achieve this goal. In that sense, different studies have called attention to the importance of increasing the number of skilled health researchers in less developed countries, for example through joint institutional training programs between countries that are more and less developed [87] or promotion of research stays [88]. Some studies have also pointed out the need to invest in infrastructure and develop the research capacity of national health systems as initial, essential steps toward ensuring a greater participation of less developed countries in scientific investigation [87,89,90].

The greater degree of participation from LHD countries in the research disseminated through mainstream journals in Tropical Medicine is doubtlessly a response to the different initiatives launched in this area and captured in the London Declaration on Neglected Tropical Diseases of 2012. With leadership from WHO, a range of institutions, governments (United States, UK, United Arab Emirate, Bangladesh, Brazil, Mozambique y Tanzania), prominent research foundations and pharmaceutical companies acknowledged the need to eradicate neglected tropical diseases [91]. As our study suggests, initiatives like these have had an important positive effect, and future research could examine this in more depth, both in Tropical Medicine as well as in other areas of special relevance to less developed countries.

The fact that studies such as Singh [92] have observed lower acceptance rates for manuscripts from MHD and LHD countries, which would encompass papers with poor English or inferior quality, also suggests the need to improve methodological and linguistic skills related to drafting and communication in the academic environment of these countries.

The order of signatures and the address for correspondence in scientific publications are bibliographic characteristics that facilitate a precise, in-depth analysis of cooperative practices and their associations with concepts like dominance, balance, and subordination with regard to participation in research activities. In that sense, collaborative papers show limited leadership and a certain subordination on the part of countries with lower HDI, presenting values that are well below those of more developed countries in relation to these bibliographic characteristics. The study of these variables has also elucidated the dominance exercised by emerging economies such as Brazil and China with regard to their cooperative links with the United States and other VHHD countries.

Scientific collaboration and the establishment of alliances with more developed countries constitute an important mechanism through which less developed countries can be integrated into research activities. However, the medium-term goal should be to empower these countries with mutually beneficial and balanced partnerships. The analysis of the order of signatures, based on the percentage of documents signed as first author, may serve as a good indicator for measuring the extent of this empowerment by MHD/LHD countries operating within North-South partnerships [86]. For example, Sewankambo et al. [87] analyzed the relationship between the Karolinska Institutet in Sweden (a VHHD country) and Makerere University in Uganda (a LHD country) using this indicator and found that 52.9% of the publications produced in collaboration featured an investigator from Makerere University as first or last author. This fact reflects a balanced association with no clear dominance or subordination from either of the two institutions, which share a joint PhD program and exchange scientific resources and insight through a partnership that may constitute a good example to follow for others.

## Supporting information

S1 Table Countries and percentage of the world population according to HDI identified in documents included in the SCI-Expanded database in the categories of Tropical Medicine, Infectious Diseases, Parasitology and Pediatrics (2011–2015). (DOCX)

S2 Table Countries and percentages of the world population group by regions and sub-regions identified in documents included in the SCI-Expanded database in the categories of Tropical Medicine, Infectious Diseases, Parasitology and Pediatrics (2011–2015). (DOCX)

S3 Table Matrix with collaboration ties and dominance indexes in Infectious Diseases publications, in documents included in the SCI-Expanded database (2011–2015). N Collaborations: ↗ Number of first authorships in collaborative documents; ↙ Number of collaborative documents without participation as lead author. Dominance indexes: ↑ Dominance index in favor of country listed in top row; ← Dominance index in favor of country included in lefthand column; = Authors from both countries have signed the same number of documents in the first position;—: no collaborative links on papers led by authors from one of the two countries.
(DOCX)

S4 Table Matrix with collaboration ties and dominance indexes in Parasitology publications, in documents included in the SCI-Expanded database (2011–2015). N Collaborations: ↗ Number of first authorships in collaborative documents; ↙ Number of collaborative documents without participation as lead author. Dominance indexes: ↑ Dominance index in favor of country listed in top row; ← Dominance index in favor of country included in lefthand column; = Authors from both countries have signed the same number of documents in the first position;—: no collaborative links on papers led by authors from one of the two countries.
(DOCX)

S5 Table Matrix with collaboration ties and dominance indexes in Pediatrics publications, in documents included in the SCI-Expanded database (2011–2015). N Collaborations: ↗ Number of first authorships in collaborative documents; ↙ Number of collaborative documents without participation as lead author. Dominance indexes: ↑ Dominance index in favor of country listed in top row; ← Dominance index in favor of country included in lefthand column; = Authors from both countries have signed the same number of documents in the first position;—: no collaborative links on papers led by authors from one of the two countries.
(DOCX)

S6 Table Number of signatures, participation as first author and corresponding author, by HDI in country of origin, in documents included in the SCI-Expanded database in the categories of Tropical Medicine, Infectious Diseases, Parasitology and Pediatrics (2011–2015). (DOCX)

S7 Table Test ANOVA for average citations per paper group by collaboration types in the documents included in SCI-Expanded database in the categories of Tropical Medicine, Infectious Diseases, Parasitology and Pediatrics (2011–2015). Diff: Mean differences between collaboration types. P-value: Significance († not significant; * p <0.05; ** p<0.01; ***p<0.001).
(DOCX)

S1 Fig Directed network generated based on the dominance indexes in the Infectious Diseases research area, in documents included in the SCI-Expanded database (2011–2015). Colors represent HDI of the countries (red: VHHD; green: HHD; blue: MHD; and yellow: LHD).
(TIF)

S2 Fig Directed network generated based on the dominance indexes in the Parasitology research area, in documents included in the SCI-Expanded database (2011–2015). Colors represent HDI of the countries (red: VHHD; green: HHD; blue: MHD; and yellow: LHD).
(TIF)

S3 Fig Directed network generated based on the dominance indexes in the Pediatrics research area, in documents included in the SCI-Expanded database (2011–2015). Colors represent HDI of the countries (red: VHHD; green: HHD; blue: MHD; and yellow: LHD).
(TIF)

S1 Appendix Countries identified with the indication of their Human Development Index (HDI) and the geographical regions and sub-regions to which they belong. (DOCX)
