# Supplementary material for: Dominance and leadership in research activities: Collaboration between countries of differing human development is reflected through authorship order and designation as corresponding authors in scientific publications
Source: PLoS One. 2017 Aug 8;12(8):e0182513. doi: 10.1371/journal.pone.0182513 (PMC5549749; doi:10.1371/journal.pone.0182513)
Supplement: S1 Table — (DOCX) [file pone.0182513.s001.docx]

**S1 Table. Countries and percentage of the world population according to HDI identified in documents included in the SCI-Expanded database in the categories of Tropical Medicine, Infectious Diseases, Parasitology and Pediatrics (2011-2015).**

| **Human Development Index** | **Tropical Medicine** | | | **Infectious Diseases** | | | **Parasitology** | | | **Pediatrics** | | |
| --- | --- | --- | --- | --- | --- | --- | --- | --- | --- | --- | --- | --- |
|  | **N countries** | **Population (millions)** | **%** | **N countries** | **Population (millions)** | **%** | **N countries** | **Population (millions)** | **%** | **N countries** | **Population (millions)** | **%** |
| Very high | 41 | 1137.59 | 17.54 | 46 | 1144.7 | 17.34 | 45 | 1143.11 | 17.54 | 46 | 1144.7 | 17.42 |
| High | 41 | 2363.53 | 36.44 | 50 | 2408.62 | 36.49 | 46 | 2375.11 | 36.44 | 47 | 2408.16 | 36.66 |
| Medium | 27 | 2059.92 | 31.76 | 38 | 2113.9 | 32.03 | 37 | 2113.8 | 32.43 | 30 | 2096.45 | 31.91 |
| Low | 39 | 924.39 | 14.25 | 44 | 932.85 | 14.13 | 43 | 886.35 | 13.6 | 38 | 919.74 | 14 |
