# Supplementary material for: Dominance and leadership in research activities: Collaboration between countries of differing human development is reflected through authorship order and designation as corresponding authors in scientific publications
Source: PLoS One. 2017 Aug 8;12(8):e0182513. doi: 10.1371/journal.pone.0182513 (PMC5549749; doi:10.1371/journal.pone.0182513)
Supplement: S2 Table — (DOCX) [file pone.0182513.s002.docx]

**S2 Table. Countries and percentages of the world population group by regions and sub-regions identified in documents included in the SCI-Expanded database in the categories of Tropical Medicine, Infectious Diseases, Parasitology and Pediatrics (2011-2015).**

| **Regions** | **Tropical Medicine** | | | **Infectious Diseases** | | | **Parasitology** | | | **Pediatrics** | | |
| --- | --- | --- | --- | --- | --- | --- | --- | --- | --- | --- | --- | --- |
|  | **N countries** | **Population (millions)** | **%** | **N countries** | **Population (millions)** | **%** | **N countries** | **Population (millions)** | **%** | **N countries** | **Population (millions)** | **%** |
| Africa | 44 | 920.21 | 14.19 | 53 | 930.46 | 14.10 | 52 | 883.96 | 13.56 | 44 | 914.06 | 13.91 |
| Asia | 39 | 3928.01 | 60.57 | 45 | 4000.89 | 60.62 | 43 | 3976.54 | 61.01 | 43 | 3987.45 | 60.70 |
| Europe | 31 | 712.27 | 10.98 | 39 | 732.88 | 11.10 | 37 | 722.41 | 11.08 | 39 | 732.88 | 11.16 |
| Latin America and the Caribbean | 26 | 548.2 | 8.45 | 30 | 558.79 | 8.47 | 29 | 558.51 | 8.57 | 28 | 558.43 | 8.50 |
| Northern America | 2 | 342.22 | 5.28 | 2 | 342.22 | 5.19 | 2 | 342.22 | 5.25 | 2 | 342.22 | 5.21 |
| Oceania | 6 | 34.52 | 0.53 | 9 | 34.83 | 0.53 | 8 | 34.72 | 0.53 | 5 | 34 | 0.52 |
| **Sub-regions** | **N countries** | **Population (millions)** | **%** | **N countries** | **Population (millions)** | **%** | **N countries** | **Population (millions)** | **%** | **N countries** | **Population (millions)** | **%** |
| Australia and New Zealand | 2 | 25.87 | 0.40 | 2 | 25.87 | 0.39 | 2 | 25.87 | 0.40 | 2 | 25.87 | 0.39 |
| Caribbean | 7 | 24.02 | 0.37 | 10 | 33.87 | 0.51 | 9 | 33.59 | 0.52 | 9 | 33.82 | 0.51 |
| Central America | 8 | 148.55 | 2.29 | 8 | 148.55 | 2.25 | 8 | 148.55 | 2.28 | 7 | 148.24 | 2.26 |
| Central Asia | 1 | 5.11 | 0.08 | 4 | 48.49 | 0.73 | 4 | 48.49 | 0.74 | 2 | 35.82 | 0.55 |
| Eastern Africa | 12 | 283 | 4.36 | 16 | 287.23 | 4.35 | 16 | 287.23 | 4.41 | 13 | 283.09 | 4.31 |
| Eastern Asia | 4 | 1539.47 | 23.74 | 5 | 1542.12 | 23.37 | 4 | 1519.01 | 23.30 | 5 | 1542.12 | 23.48 |
| Eastern Europe | 8 | 282.9 | 4.36 | 10 | 295.79 | 4.48 | 9 | 286.28 | 4.39 | 10 | 295.79 | 4.50 |
| Melanesia | 4 | 8.65 | 0.13 | 4 | 8.65 | 0.13 | 4 | 8.65 | 0.13 | 3 | 8.13 | 0.12 |
| Micronesia | - | - | - | 2 | 0.2 | 0.00 | 1 | 0.1 | 0.00 | - | - | - |
| Middle Africa | 8 | 137.15 | 2.11 | 10 | 138.35 | 2.10 | 9 | 91.85 | 1.41 | 8 | 137.15 | 2.09 |
| Northern Africa | 6 | 160.76 | 2.48 | 6 | 160.76 | 2.44 | 6 | 160.76 | 2.47 | 6 | 160.76 | 2.45 |
| Northern America | 2 | 342.22 | 5.28 | 2 | 342.22 | 5.19 | 2 | 342.22 | 5.25 | 2 | 342.22 | 5.21 |
| Northern Europe | 6 | 92.96 | 1.43 | 10 | 99.65 | 1.51 | 10 | 99.65 | 1.53 | 10 | 99.65 | 1.52 |
| Polynesia | - | - | - | 1 | 0.1 | 0.00 | 1 | 0.1 | 0.00 | - | - | - |
| South America | 11 | 375.62 | 5.79 | 12 | 376.37 | 5.70 | 12 | 376.37 | 5.77 | 12 | 376.37 | 5.73 |
| South-Eastern Asia | 11 | 584.6 | 9.01 | 11 | 584.6 | 8.86 | 11 | 584.6 | 8.97 | 10 | 583.53 | 8.88 |
| Southern Africa | 4 | 57.65 | 0.89 | 5 | 58.49 | 0.89 | 5 | 58.49 | 0.90 | 4 | 56.38 | 0.86 |
| Southern Asia | 8 | 1621.07 | 25.00 | 8 | 1621.07 | 24.56 | 8 | 1621.07 | 24.87 | 9 | 1621.37 | 24.68 |
| Southern Europe | 10 | 150.08 | 2.31 | 12 | 151.11 | 2.29 | 11 | 150.14 | 2.30 | 12 | 151.11 | 2.30 |
| Western Africa | 14 | 281.66 | 4.34 | 16 | 285.63 | 4.33 | 16 | 285.63 | 4.38 | 13 | 276.68 | 4.21 |
| Western Asia | 15 | 177.75 | 2.74 | 17 | 204.6 | 3.10 | 16 | 203.36 | 3.12 | 17 | 204.6 | 3.11 |
| Western Europe | 7 | 186.34 | 2.87 | 7 | 186.34 | 2.82 | 7 | 186.34 | 2.86 | 7 | 186.34 | 2.84 |
