# Supplementary material for: Dominance and leadership in research activities: Collaboration between countries of differing human development is reflected through authorship order and designation as corresponding authors in scientific publications
Source: PLoS One. 2017 Aug 8;12(8):e0182513. doi: 10.1371/journal.pone.0182513 (PMC5549749; doi:10.1371/journal.pone.0182513)
Supplement: S3 Table — N Collaborations: ↗ Number of first authorships in collaborative documents; ↙ Number of collaborative documents without participation as lead author. Dominance indexes: ↑ Dominance index in favor of country listed in top row; ← Dominance index in favor of country included in lefthand column; = Authors from both countries have signed the same number of documents in the first position;—: no collaborative links on papers led by authors from one of the two countries. (DOCX) [file pone.0182513.s003.docx]

**S3 Table. Matrix with collaboration ties and dominance indexes in Infectious Diseases publications, in documents included in the SCI-Expanded database (2011-2015).**

| Dominance Indexes  N collaborations | Australia | Brazil | France | Germany | India | Japan | Nigeria | Pakistan | China | South Africa | UK | USA |
| --- | --- | --- | --- | --- | --- | --- | --- | --- | --- | --- | --- | --- |
| Australia |  | 🡩0.66 | 🡨0.54 | 🡩0.65 | = | 🡨0.71 | 🡨0.8 | 🡨0.67 | 🡩0.53 | 🡩0.55 | 🡨0.54 | 🡩0.54 |
| Brazil | 🡭21 🡯11 |  | 🡨0.66 | 🡩0.56 | 🡨0.57 | 🡨0.7 | 🡨0.75 | = | 🡩0.57 | 🡩0.53 | 🡨0.61 | 🡨0.58 |
| France | 🡭41 🡯49 | 🡭23 🡯44 |  | 🡩0.54 | 🡨0.52 | 🡨0.76 | 🡨0.73 | 🡩0.67 | 🡩0.67 | 🡩0.56 | 🡩0.61 | 🡩0.55 |
| Germany | 🡭18 🡯34 | 🡭23 🡯18 | 🡭83 🡯70 |  | 🡨0.59 | 🡨0.63 | 🡨0.59 | = | 🡩0.58 | 🡩0.67 | 🡩0.55 | 🡩0.53 |
| India | 🡭19 🡯19 | 🡭3 🡯4 | 🡭18 🡯20 | 🡭10 🡯12 |  | 🡨0.69 | = | = | 🡨0.78 | 🡨0.55 | 🡩0.66 | 🡩0.6 |
| Japan | 🡭7 🡯17 | 🡭3 🡯7 | 🡭4 🡯13 | 🡭7 🡯12 | 🡭7 🡯16 |  | = | = | 🡩0.63 | 🡩0.75 | 🡨0.53 | 🡨0.58 |
| Nigeria | 🡭1 🡯4 | 🡭1 🡯3 | 🡭3 🡯8 | 🡭7 🡯10 | 🡭1 🡯1 | 🡭1 🡯1 |  | 🡩1 | = | = | 🡩0.59 | 🡩0.64 |
| Pakistan | 🡭2 🡯4 | 🡭1 🡯1 | 🡭6 🡯3 | 🡭2 🡯2 | 🡭2 🡯2 | 🡭1 🡯1 | 🡭1 🡯0 |  | 🡩0.54 | 🡨1 | 🡨0.53 | 🡩0.61 |
| China | 🡭70 🡯61 | 🡭4 🡯3 | 🡭28 🡯14 | 🡭18 🡯13 | 🡭7 🡯2 | 🡭52 🡯31 | 🡭2 🡯2 | 🡭6 🡯5 |  | = | 🡨0.7 | 🡨0.65 |
| South Africa | 🡭27 🡯22 | 🡭8 🡯7 | 🡭34 🡯27 | 🡭29 🡯14 | 🡭8 🡯10 | 🡭3 🡯1 | 🡭10 🡯10 | 🡭0 🡯3 | 🡭4 🡯4 |  | 🡩0.52 | 🡩0.6 |
| UK | 🡭159 🡯186 | 🡭41 🡯64 | 🡭208 🡯133 | 🡭159 🡯127 | 🡭53 🡯27 | 🡭15 🡯17 | 🡭27 🡯19 | 🡭15 🡯17 | 🡭37 🡯88 | 🡭179 🡯165 |  | 🡩0.54 |
| USA | 🡭608 🡯515 | 🡭238 🡯333 | 🡭238 🡯195 | 🡭177 🡯159 | 🡭196 🡯130 | 🡭83 🡯115 | 🡭78 🡯44 | 🡭39 🡯25 | 🡭262 🡯480 | 🡭470 🡯312 | 🡭608 🡯515 |  |

N Collaborations: 🡭 Number of first authorships in collaborative documents; 🡯 Number of collaborative documents without participation as lead author. Dominance indexes: 🡩 Dominance index in favor of country listed in top row; 🡨 Dominance index in favor of country included in lefthand column; = Authors from both countries have signed the same number of documents in the first position; — : no collaborative links on papers led by authors from one of the two countries.
