# Supplementary material for: Dominance and leadership in research activities: Collaboration between countries of differing human development is reflected through authorship order and designation as corresponding authors in scientific publications
Source: PLoS One. 2017 Aug 8;12(8):e0182513. doi: 10.1371/journal.pone.0182513 (PMC5549749; doi:10.1371/journal.pone.0182513)
Supplement: S4 Table — N Collaborations: ↗ Number of first authorships in collaborative documents; ↙ Number of collaborative documents without participation as lead author. Dominance indexes: ↑ Dominance index in favor of country listed in top row; ← Dominance index in favor of country included in lefthand column; = Authors from both countries have signed the same number of documents in the first position;—: no collaborative links on papers led by authors from one of the two countries. (DOCX) [file pone.0182513.s004.docx]

**S4 Table. Matrix with collaboration ties and dominance indexes in Parasitology publications, in documents included in the SCI-Expanded database (2011-2015).**

| Dominance Indexes  N collaborations | Australia | Brazil | France | Germany | India | Japan | Nigeria | Pakistan | China | South Africa | UK | USA |
| --- | --- | --- | --- | --- | --- | --- | --- | --- | --- | --- | --- | --- |
| Australia |  | 🡩0.69 | 🡩0.53 | = | 🡨0.64 | 🡨0.64 | 🡩0.67 | 🡩0.62 | 🡩0.65 | 🡩0.55 | 🡨0.59 | 🡩0.51 |
| Brazil | 🡭18 🡯8 |  | 🡨0.8 | 🡨0.53 | 🡩1 | 🡨0.75 | 🡩0.67 | 🡩1 | 🡩0.67 | 🡨1 | 🡨0.76 | 🡨0.7 |
| France | 🡭17 🡯15 | 🡭11 🡯41 |  | 🡩0.52 | 🡩0.78 | 🡨0.56 | = | 🡩0.8 | 🡩0.7 | 🡨0.69 | 🡨0.55 | 🡩0.5 |
| Germany | 🡭17 🡯17 | 🡭23 🡯26 | 🡭51 🡯47 |  | 🡩0.6 | 🡩0.61 | = | 🡩1 | 🡩0.64 | 🡨0.58 | 🡨0.55 | 🡨0.55 |
| India | 🡭9 🡯5 | 🡭1 🡯0 | 🡭14 🡯4 | 🡭12 🡯8 |  | 🡩0.75 | 🡩1 | — | 🡨0.57 | — | 🡩0.55 | 🡨0.54 |
| Japan | 🡭9 🡯5 | 🡭3 🡯9 | 🡭10 🡯13 | 🡭8 🡯5 | 🡭4 🡯12 |  | 🡨0.6 | 🡨0.67 | 🡩0.51 | 🡨1 | 🡩0.54 | 🡩0.6 |
| Nigeria | 🡭2 🡯1 | 🡭2 🡯1 | 🡭1 🡯1 | 🡭3 🡯3 | 🡭2 🡯0 | 🡭2 🡯3 |  | — | 🡩1 | 🡨0.67 | 🡩0.61 | 🡩0.67 |
| Pakistan | 🡭5 🡯3 | 🡭0 🡯1 | 🡭4 🡯1 | 🡭1 🡯0 | 🡭0 🡯0 | 🡭1 🡯2 | 🡭0 🡯0 |  | 🡩0.67 | 🡨1 | 🡨0.7 | 🡨0.75 |
| China | 🡭49 🡯26 | 🡭4 🡯2 | 🡭26 🡯11 | 🡭16 🡯9 | 🡭3 🡯4 | 🡭28 🡯27 | 🡭2 🡯0 | 🡭4 🡯2 |  | 🡨0.75 | 🡨0.72 | 🡨0.68 |
| South Africa | 🡭11 🡯9 | 🡭0 🡯5 | 🡭13 🡯29 | 🡭19 🡯26 | 🡭0 🡯0 | 🡭0 🡯4 | 🡭1 🡯2 | 🡭0 🡯1 | 🡭2 🡯6 |  | 🡩0.71 | 🡩0.63 |
| UK | 🡭71 🡯104 | 🡭29 🡯91 | 🡭78 🡯96 | 🡭67 🡯81 | 🡭23 🡯19 | 🡭20 🡯17 | 🡭20 🡯13 | 🡭5 🡯12 | 🡭22 🡯56 | 🡭63 🡯26 |  | 🡩0.51 |
| USA | 🡭105 🡯99 | 🡭119 🡯281 | 🡭101 🡯100 | 🡭93 🡯115 | 🡭50 🡯60 | 🡭62 🡯41 | 🡭27 🡯13 | 🡭5 🡯15 | 🡭105 🡯228 | 🡭45 🡯26 | 🡭273 🡯283 |  |

N Collaborations: 🡭 Number of first authorships in collaborative documents; 🡯 Number of collaborative documents without participation as lead author. Dominance indexes: 🡩 Dominance index in favor of country listed in top row; 🡨 Dominance index in favor of country included in lefthand column; = Authors from both countries have signed the same number of documents in the first position; — : no collaborative links on papers led by authors from one of the two countries.
