# Supplementary material for: Dominance and leadership in research activities: Collaboration between countries of differing human development is reflected through authorship order and designation as corresponding authors in scientific publications
Source: PLoS One. 2017 Aug 8;12(8):e0182513. doi: 10.1371/journal.pone.0182513 (PMC5549749; doi:10.1371/journal.pone.0182513)
Supplement: S5 Table — N Collaborations: ↗ Number of first authorships in collaborative documents; ↙ Number of collaborative documents without participation as lead author. Dominance indexes: ↑ Dominance index in favor of country listed in top row; ← Dominance index in favor of country included in lefthand column; = Authors from both countries have signed the same number of documents in the first position;—: no collaborative links on papers led by authors from one of the two countries. (DOCX) [file pone.0182513.s005.docx]

**S5 Table. Matrix with collaboration ties and dominance indexes in Pediatrics publications, in documents included in the SCI-Expanded database (2011-2015).**

| Dominance Indexes  N collaborations | Australia | Brazil | France | Germany | India | Japan | Nigeria | Pakistan | China | South Africa | UK | USA |
| --- | --- | --- | --- | --- | --- | --- | --- | --- | --- | --- | --- | --- |
| Australia |  | 🡩0.53 | = | = | 🡩0.81 | 🡨0.7 | 🡨1 | = | 🡩0.55 | 🡩0.72 | 🡨0.64 | 🡩0.54 |
| Brazil | 🡭9 🡯8 |  | 🡩0.55 | 🡨0.6 | 🡨1 | — | 🡩1 | 🡨1 | 🡨1 | 🡩1 | 🡨0.71 | 🡨0.54 |
| France | 🡭11 🡯11 | 🡭11 🡯9 |  | 🡩0.58 | 🡩0.77 | = | — | — | 🡩0.57 | = | 🡩0.5 | 🡩0.54 |
| Germany | 🡭28 🡯28 | 🡭4 🡯6 | 🡭41 🡯29 |  | 🡩0.76 | 🡨0.7 | — | 🡨0.67 | 🡨0.58 | 🡨0.62 | 🡨0.56 | 🡩0.51 |
| India | 🡭21 🡯5 | 🡭0 🡯3 | 🡭10 🡯3 | 🡭13 🡯4 |  | 🡩1 | 🡩0.67 | = | 🡨0.71 | = | 🡨0.58 | 🡩0.51 |
| Japan | 🡭3 🡯7 | 🡭0 🡯0 | 🡭1 🡯1 | 🡭3 🡯7 | 🡭0 🡯5 |  | 🡨1 | — | 🡩0.61 | = | 🡨0.7 | 🡩0.57 |
| Nigeria | 🡭0 🡯1 | 🡭1 🡯0 | 🡭0 🡯0 | 🡭0 🡯0 | 🡭4 🡯2 | 🡭0 🡯1 |  | — | = | 🡨0.54 | 🡨0.8 | 🡩0.65 |
| Pakistan | 🡭2 🡯2 | 🡭0 🡯2 | 🡭0 🡯0 | 🡭1 🡯2 | 🡭1 🡯1 | 🡭0 🡯0 | 🡭0 🡯0 |  | — | — | = | 🡩0.55 |
| China | 🡭41 🡯33 | 🡭0 🡯3 | 🡭3 🡯4 | 🡭5 🡯7 | 🡭2 🡯5 | 🡭11 🡯7 | 🡭1 🡯1 | 🡭0 🡯0 |  | = | 0.56 | 🡨0.58 |
| South Africa | 🡭13 🡯5 | 🡭2 🡯0 | 🡭2 🡯2 | 🡭3 🡯5 | 🡭1 🡯1 | 🡭1 🡯1 | 🡭5 🡯6 | 🡭0 🡯0 | 🡭1 🡯1 |  | 🡨0.56 | 🡩0.51 |
| UK | 🡭108 🡯191 | 🡭14 🡯35 | 🡭58 🡯57 | 🡭75 🡯97 | 🡭24 🡯33 | 🡭3 🡯7 | 🡭2 🡯8 | 🡭11 🡯11 | 🡭15 🡯19 | 🡭24 🡯31 |  | 🡩0.62 |
| USA | 🡭174 🡯150 | 🡭86 🡯100 | 🡭80 🡯67 | 🡭135 🡯130 | 🡭80 🡯76 | 🡭58 🡯44 | 🡭22 🡯12 | 🡭20 🡯16 | 🡭116 🡯162 | 🡭62 🡯60 | 🡭328 🡯200 |  |

N Collaborations: 🡭 Number of first authorships in collaborative documents; 🡯 Number of collaborative documents without participation as lead author. Dominance indexes: 🡩 Dominance index in favor of country listed in top row; 🡨 Dominance index in favor of country included in lefthand column; = Authors from both countries have signed the same number of documents in the first position; — : no collaborative links on papers led by authors from one of the two countries.
