# Supplementary material for: Dominance and leadership in research activities: Collaboration between countries of differing human development is reflected through authorship order and designation as corresponding authors in scientific publications
Source: PLoS One. 2017 Aug 8;12(8):e0182513. doi: 10.1371/journal.pone.0182513 (PMC5549749; doi:10.1371/journal.pone.0182513)
Supplement: S6 Table — (DOCX) [file pone.0182513.s006.docx]

**S6 Table. Number of signatures, participation as first author and corresponding author, by HDI in country of origin, in documents included in the SCI-Expanded database in the categories of Tropical Medicine, Infectious Diseases, Parasitology and Pediatrics (2011-2015).**

| **Research Area** | | **Very high HDI** | **High HDI** | **Medium HDI** | **Low HDI** |
| --- | --- | --- | --- | --- | --- |
|  |  | **N docs (%)** | **N docs (%)** | **N docs (%)** | **N docs (%)** |
| Tropical Medicine | Total signatures | 13,226 (46.27) | 7675 (26.85) | 3697 (12.93) | 3983 (13.93) |
|  | 1^st^ position | 6245 (38.45) | 6321 (38.92) | 2166 (13.33) | 1508 (9.28) |
|  | Corresponding author | 6522 (40.23) | 6265 (38.65) | 2070 (12.77) | 1353 (8.35) |
| Infectious Diseases | Total signatures | 73,390 (71.61) | 14,658 (14.3) | 7566 (7.38) | 6865 (6.7) |
|  | 1^st^ position | 48,279 (74.44) | 10,627 (16.38) | 3667 (5.65) | 2286 (3.52) |
|  | Corresponding author | 48,898 (75.45) | 10,407 (16.06) | 3459 (5.34) | 2040 (3.15) |
| Parasitology | Total signatures | 28,638 (61.25) | 10,570 (22.61) | 3926 (8.4) | 3619 (7.74) |
|  | 1^st^ position | 16,562 (58.21) | 8329 (29.27) | 2144 (7.54) | 1415 (4.97) |
|  | Corresponding author | 16,844 (59.04) | 8337 (29.22) | 2031 (7.12) | 1316 (4.61) |
| Pediatrics | Total signatures | 75,430 (81.25) | 11,318 (12.19) | 4871 (5.25) | 1220 (1.31) |
|  | 1^st^ position | 61,415 (80.83) | 9953 (13.1) | 3999 (5.26) | 612 (0.8) |
|  | Corresponding author | 61,657 (81.17) | 9810 (12.91) | 3928 (5.17) | 561 (0.74) |

N docs: Number of documents.
