# Supplementary material for: Dominance and leadership in research activities: Collaboration between countries of differing human development is reflected through authorship order and designation as corresponding authors in scientific publications
Source: PLoS One. 2017 Aug 8;12(8):e0182513. doi: 10.1371/journal.pone.0182513 (PMC5549749; doi:10.1371/journal.pone.0182513)
Supplement: S7 Table — Diff: Mean differences between collaboration types. P-value: Significance († not significant; * p <0.05; ** p<0.01; ***p<0.001). (DOCX) [file pone.0182513.s007.docx]

**S7 Table. Test ANOVA for average citations per paper group by collaboration types in the documents included in SCI-Expanded database in the categories of Tropical Medicine, Infectious Diseases, Parasitology and Pediatrics (2011-2015).**

| **Collaboration types** | **Tropical Medicine** | | **Infectious Diseases** | | **Parasitology** | | **Pediatrics** | |
| --- | --- | --- | --- | --- | --- | --- | --- | --- |
|  | Diff | P-value | Diff | P-value | Diff | P-value | Diff | P-value |
| 2-1 | .7273^**^ | .009 | .2962† | .726 | -.1156† | .999 | **.7924^***^** | **.000** |
| 3-1 | -1.0877^*^ | .030 | **-3.5029^***^** | **.000** | **-3.0447^***^** | **.000** | **-1.5427^***^** | **.000** |
| 4-1 | -.8398^*^ | .048 | **-3.2278^***^** | **.000** | **-3.0447^***^** | **.000** | **-1.4760^***^** | **.000** |
| 5-1 | **2.0293^***^** | **.000** | .6219† | .955 | -1.0015† | .610 | .3422† | .999 |
| 6-1 | 1.2983^*^ | .029 | -.4346† | .999 | -1.0935† | .461 | -.3334† | .996 |
| 7-1 | **3.0661^***^** | **.000** | **1.6879^***^** | **.000** | .3443† | .950 | **1.3875^***^** | **.000** |
| 8-1 | **1.7834^***^** | **.000** | -.3397† | .967 | -.9902† | .155 | .1385† | 1.000 |
| 3-2 | **-1.8150^***^** | **.000** | **3.7991^***^** | **.000** | **-2.9292^***^** | **.000** | **-2.3352^***^** | **.000** |
| 4-2 | **-1.5672^***^** | **.000** | **-3.5239^***^** | **.000** | **-2.9156^***^** | **.000** | **-2.2685^***^** | **.000** |
| 5-2 | 1.3020^*^ | .010 | .3258† | .999 | -.8859† | .688 | -.4502† | .992 |
| 6-2 | .5709† | .776 | -.7308† | .973 | -.9779† | .531 | -1.1258† | .213 |
| 7-2 | **2.3388^***^** | **.000** | **1.3917^***^** | **.000** | .4598† | .562 | .5950† | .275 |
| 8-2 | **1.0561^***^** | **.000** | -.6359† | .339 | -.8746† | .150 | -.6539† | .521 |
| 4-3 | .2479† | .997 | .2752† | 1.000 | .0135† | 1.000 | .0667† | 1.000 |
| 5-3 | **3.1170^***^** | **.000** | **4.1249^***^** | **.000** | 2.0432^*^ | .050 | 1.8850^*^ | .026 |
| 6-3 | **2.3859^***^** | **.000** | 3.0683^*^ | .021 | 1.9512† | .064 | 1.2094† | .229 |
| 7-3 | **4.1538^***^** | **.000** | **5.1908^***^** | **.000** | **3.3890^***^** | **.000** | **2.9302^***^** | **.000** |
| 8-3 | **2.8711^***^** | **.000** | **3.1632^***^** | **.000** | 2.0545^**^ | .004 | **1.6812^***^** | **.000** |
| 5-4 | **2.8691^***^** | **.000** | **3.8497^***^** | **.000** | 2.0297^*^ | .017 | 1.8183^*^ | .029 |
| 6-4 | **2.1381^***^** | **.000** | 2.7931^*^ | .010 | 1.9377^*^ | .023 | 1.1427† | .253 |
| 7-4 | **3.9059^***^** | **.000** | **4.9156^***^** | **.000** | **3.3755^***^** | **.000** | **2.8635^***^** | **.000** |
| 8-4 | **2.6232^***^** | **.000** | **2.8880^***^** | **.000** | **2.0410^***^** | **.000** | **1.6145^***^** | **.000** |
| 6-5 | -.7311† | .837 | -1.0566† | .939 | -.0920† | 1.000 | -.6756† | .981 |
| 7-5 | 1.0368† | .139 | 1.0659† | .586 | 1.3458† | .249 | 1.0452† | .663 |
| 8-5 | -.2459† | .999 | -.9617† | .755 | .0113† | 1.000 | -.2037† | 1.000 |
| 7-6 | **1.7679^****^** | **.000** | 2.1225† | .090 | 1.4378† | .153 | 1.7208^*^ | .022 |
| 8-6 | .4852† | .942 | .0949† | 1.000 | .1033† | 1.000 | .4718† | .991 |
| 8-7 | **-1.2827^***^** | **.000** | **-2.0276^***^** | **.000** | -1.3345^*^ | .018 | -1.2490† | .059 |

Diff: Mean differences between collaboration types. P-value: Significance († not significant; * p <0.05; ** p<0.01; ***p<0.001).
