# Supplementary figures and images for: Dominance and leadership in research activities: Collaboration between countries of differing human development is reflected through authorship order and designation as corresponding authors in scientific publications
Source: PLoS One. 2017 Aug 8;12(8):e0182513. doi: 10.1371/journal.pone.0182513 (PMC5549749; doi:10.1371/journal.pone.0182513)

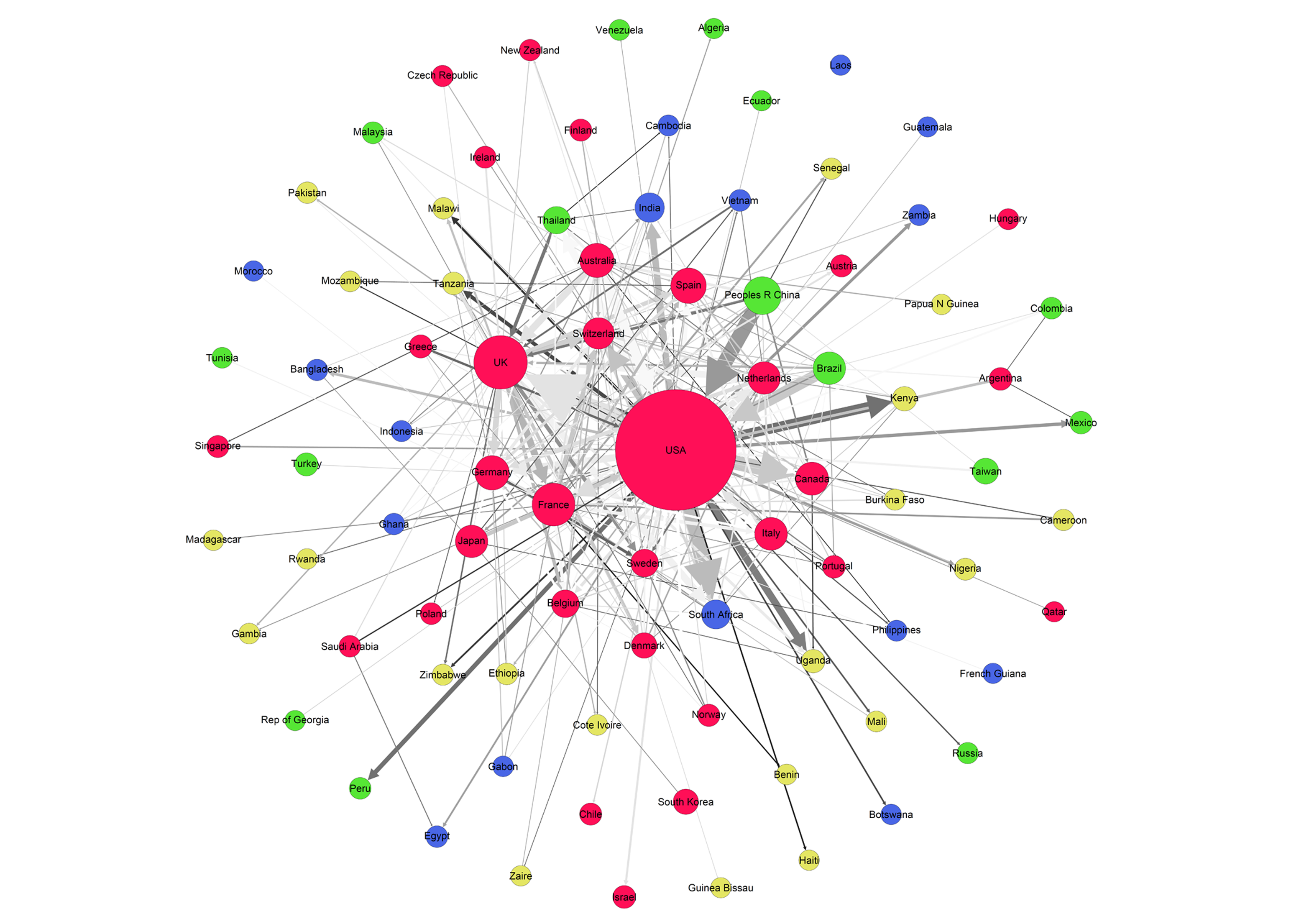

Supplement: S1 Fig — Colors represent HDI of the countries (red: VHHD; green: HHD; blue: MHD; and yellow: LHD). (TIF) [file pone.0182513.s008.tif]

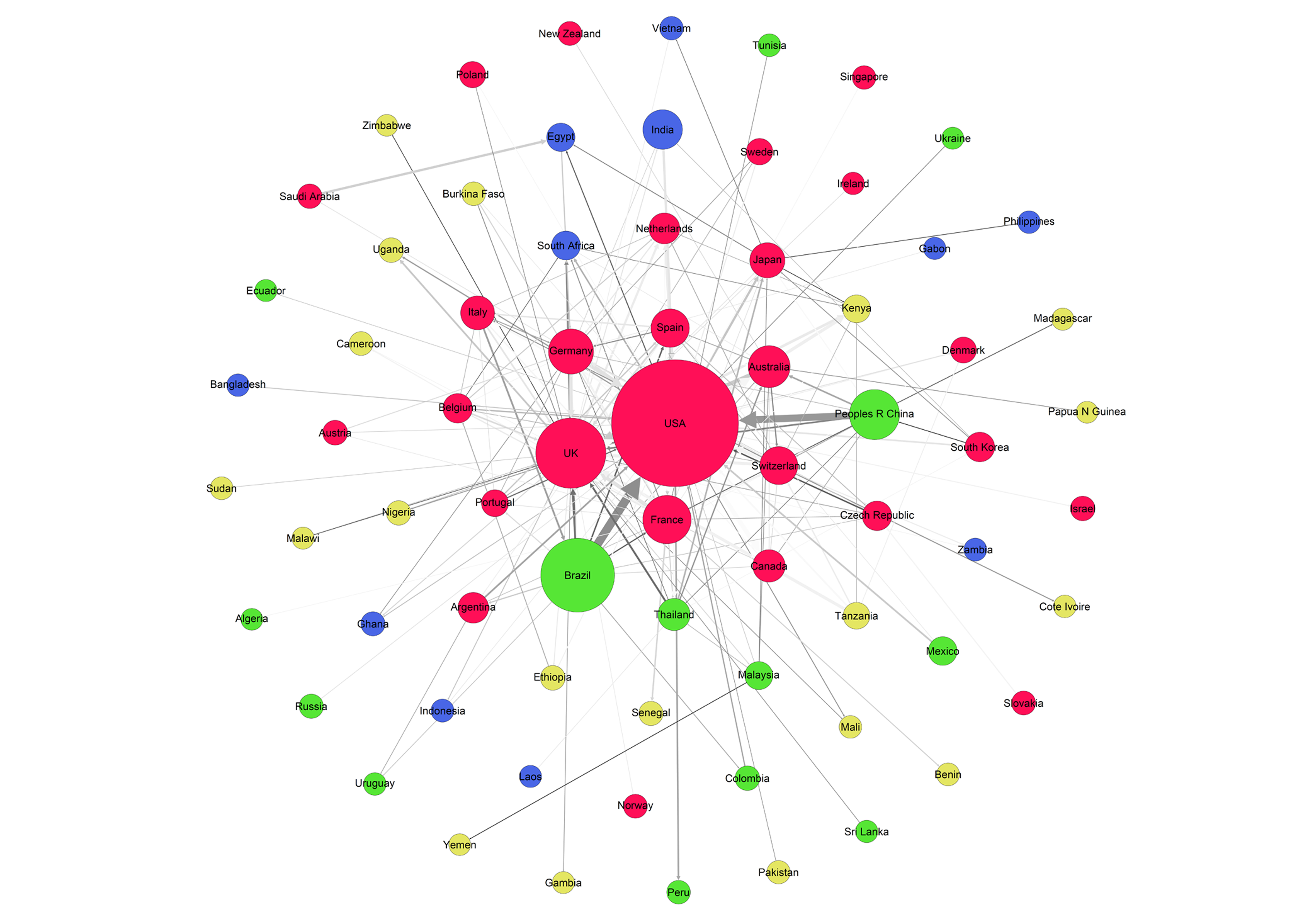

Supplement: S2 Fig — Colors represent HDI of the countries (red: VHHD; green: HHD; blue: MHD; and yellow: LHD). (TIF) [file pone.0182513.s009.tif]

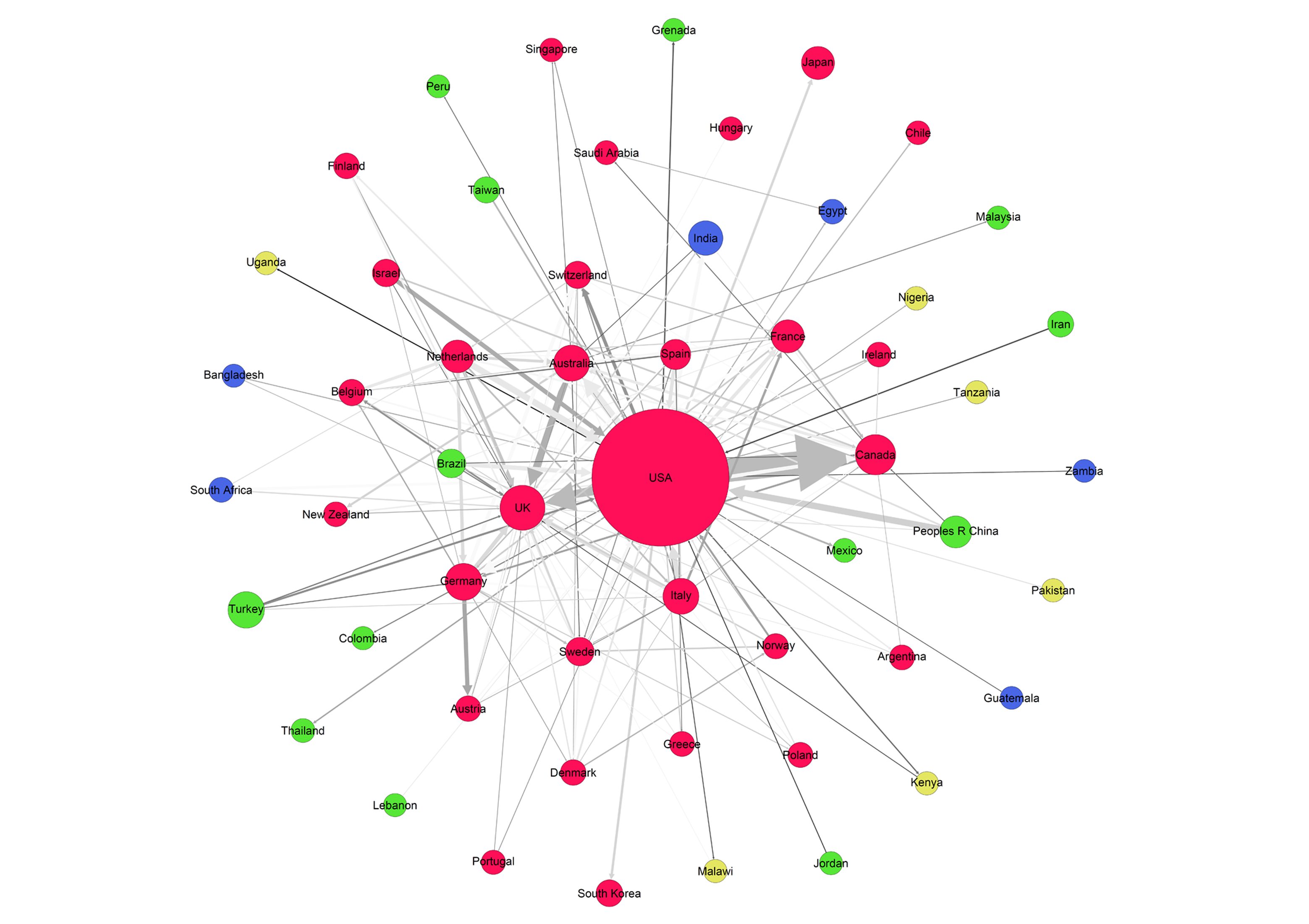

Supplement: S3 Fig — Colors represent HDI of the countries (red: VHHD; green: HHD; blue: MHD; and yellow: LHD). (TIF) [file pone.0182513.s010.tif]
