# Supplementary material for: Dominance and leadership in research activities: Collaboration between countries of differing human development is reflected through authorship order and designation as corresponding authors in scientific publications
Source: PLoS One. 2017 Aug 8;12(8):e0182513. doi: 10.1371/journal.pone.0182513 (PMC5549749; doi:10.1371/journal.pone.0182513)
Supplement: S1 Appendix — (DOCX) [file pone.0182513.s011.docx]

**S1 Appendix. Countries identified with the indication of their Human Development Index (HDI) and the geographical regions and sub-regions to which they belong.**

| **Country*** | **HDI (value)** | **HDI (category)** | **Geographical region** | **Geographical sub-region** |
| --- | --- | --- | --- | --- |
| Afghanistan | 0,468 | Low HDI | Asia | Southern Asia |
| Albania | 0,716 | High HDI | Europe | Southern Europe |
| Algeria | 0,717 | High HDI | Africa | Northern Africa |
| Andorra | 0,830 | Very high HDI | Europe | Southern Europe |
| Angola | 0,526 | Low HDI | Africa | Middle Africa |
| Antigua and Barbuda | 0,774 | High HDI | Latin America and the Caribbean | Caribbean |
| Argentina | 0,808 | Very high HDI | Latin America and the Caribbean | South America |
| Armenia | 0,730 | High HDI | Asia | Western Asia |
| Australia | 0,933 | Very high HDI | Oceania | Australia and New Zealand |
| Austria | 0,881 | Very high HDI | Europe | Western Europe |
| Azerbaijan | 0,747 | High HDI | Asia | Western Asia |
| Bahamas | 0,789 | High HDI | Latin America and the Caribbean | Caribbean |
| Bahrain | 0,815 | Very high HDI | Asia | Western Asia |
| Bangladesh | 0,558 | Medium HDI | Asia | Southern Asia |
| Barbados | 0,776 | High HDI | Latin America and the Caribbean | Caribbean |
| Belarus | 0,786 | High HDI | Europe | Eastern Europe |
| Belgium | 0,881 | Very high HDI | Europe | Western Europe |
| Belize | 0,732 | High HDI | Latin America and the Caribbean | Central America |
| Benin | 0,476 | Low HDI | Africa | Western Africa |
| Bhutan | 0,584 | Medium HDI | Asia | Southern Asia |
| Bolivia (Plurinational State of) | 0,667 | Medium HDI | Latin America and the Caribbean | South America |
| Bosnia and Herzegovina | 0,731 | High HDI | Europe | Southern Europe |
| Botswana | 0,683 | Medium HDI | Africa | Southern Africa |
| Brazil | 0,744 | High HDI | Latin America and the Caribbean | South America |
| Brunei Darussalam | 0,852 | Very high HDI | Asia | South-Eastern Asia |
| Bulgaria | 0,777 | High HDI | Europe | Eastern Europe |
| Burkina Faso | 0,388 | Low HDI | Africa | Western Africa |
| Burundi | 0,389 | Low HDI | Africa | Eastern Africa |
| Cambodia | 0,584 | Medium HDI | Asia | South-Eastern Asia |
| Cameroon | 0,504 | Low HDI | Africa | Middle Africa |
| Canada | 0,902 | Very high HDI | Northern America | Northern America |
| Cape Verde | 0,636 | Medium HDI | Africa | Western Africa |
| Central African Republic | 0,341 | Low HDI | Africa | Middle Africa |
| Chad | 0,372 | Low HDI | Africa | Middle Africa |
| Chile | 0,822 | Very high HDI | Latin America and the Caribbean | South America |
| China | 0,719 | High HDI | Asia | Eastern Asia |
| Colombia | 0,711 | High HDI | Latin America and the Caribbean | South America |
| Comoros | 0,488 | Low HDI | Africa | Eastern Africa |
| Congo | 0,564 | Medium HDI | Africa | Middle Africa |
| Congo (Democratic Republic of the) | 0,338 | Low HDI | Africa | Middle Africa |
| Costa Rica | 0,763 | High HDI | Latin America and the Caribbean | Central America |
| Côte d'Ivoire | 0,452 | Low HDI | Africa | Western Africa |
| Croatia | 0,812 | Very high HDI | Europe | Southern Europe |
| Cuba | 0,815 | Very high HDI | Latin America and the Caribbean | Caribbean |
| Cyprus | 0,845 | Very high HDI | Asia | Western Asia |
| Czech Republic | 0,861 | Very high HDI | Europe | Eastern Europe |
| Denmark | 0,900 | Very high HDI | Europe | Northern Europe |
| Djibouti | 0,467 | Low HDI | Africa | Eastern Africa |
| Dominica | 0,717 | High HDI | Latin America and the Caribbean | Caribbean |
| Dominican Republic | 0,700 | High HDI | Latin America and the Caribbean | Caribbean |
| Ecuador | 0,711 | High HDI | Latin America and the Caribbean | South America |
| Egypt | 0,682 | Medium HDI | Africa | Northern Africa |
| El Salvador | 0,662 | Medium HDI | Latin America and the Caribbean | Central America |
| Equatorial Guinea | 0,556 | Medium HDI | Africa | Middle Africa |
| Eritrea | 0,381 | Low HDI | Africa | Eastern Africa |
| Estonia | 0,840 | Very high HDI | Europe | Northern Europe |
| Ethiopia | 0,435 | Low HDI | Africa | Eastern Africa |
| Fiji | 0,724 | High HDI | Oceania | Melanesia |
| Finland | 0,879 | Very high HDI | Europe | Northern Europe |
| France | 0,884 | Very high HDI | Europe | Western Europe |
| Gabon | 0,674 | Medium HDI | Africa | Middle Africa |
| Gambia | 0,441 | Low HDI | Africa | Western Africa |
| Georgia | 0,744 | High HDI | Asia | Western Asia |
| Germany | 0,911 | Very high HDI | Europe | Western Europe |
| Ghana | 0,573 | Medium HDI | Africa | Western Africa |
| Greece | 0,853 | Very high HDI | Europe | Southern Europe |
| Grenada | 0,744 | High HDI | Latin America and the Caribbean | Caribbean |
| Guatemala | 0,628 | Medium HDI | Latin America and the Caribbean | Central America |
| Guinea | 0,392 | Low HDI | Africa | Western Africa |
| Guinea-Bissau | 0,396 | Low HDI | Africa | Western Africa |
| Guyana | 0,638 | Medium HDI | Latin America and the Caribbean | South America |
| Haiti | 0,471 | Low HDI | Latin America and the Caribbean | Caribbean |
| Honduras | 0,617 | Medium HDI | Latin America and the Caribbean | Central America |
| Hong Kong, China (SAR) | 0,891 | Very high HDI | Asia | Eastern Asia |
| Hungary | 0,818 | Very high HDI | Europe | Eastern Europe |
| Iceland | 0,895 | Very high HDI | Europe | Northern Europe |
| India | 0,586 | Medium HDI | Asia | Southern Asia |
| Indonesia | 0,684 | Medium HDI | Asia | South-Eastern Asia |
| Iran (Islamic Republic of) | 0,749 | High HDI | Asia | Southern Asia |
| Iraq | 0,642 | Medium HDI | Asia | Western Asia |
| Ireland | 0,899 | Very high HDI | Europe | Northern Europe |
| Israel | 0,888 | Very high HDI | Asia | Western Asia |
| Italy | 0,872 | Very high HDI | Europe | Southern Europe |
| Jamaica | 0,715 | High HDI | Latin America and the Caribbean | Caribbean |
| Japan | 0,890 | Very high HDI | Asia | Eastern Asia |
| Jordan | 0,745 | High HDI | Asia | Western Asia |
| Kazakhstan | 0,757 | High HDI | Asia | Central Asia |
| Kenya | 0,535 | Low HDI | Africa | Eastern Africa |
| Kiribati | 0,607 | Medium HDI | Oceania | Micronesia |
| Korea (Republic of) | 0,891 | Very high HDI | Asia | Eastern Asia |
| Kuwait | 0,814 | Very high HDI | Asia | Western Asia |
| Kyrgyzstan | 0,628 | Medium HDI | Asia | Central Asia |
| Lao People's Democratic Republic | 0,569 | Medium HDI | Asia | South-Eastern Asia |
| Latvia | 0,810 | Very high HDI | Europe | Northern Europe |
| Lebanon | 0,765 | High HDI | Asia | Western Asia |
| Lesotho | 0,486 | Low HDI | Africa | Southern Africa |
| Liberia | 0,412 | Low HDI | Africa | Western Africa |
| Libya | 0,784 | High HDI | Africa | Northern Africa |
| Liechtenstein | 0,889 | Very high HDI | Europe | Western Europe |
| Lithuania | 0,834 | Very high HDI | Europe | Northern Europe |
| Luxembourg | 0,881 | Very high HDI | Europe | Western Europe |
| Madagascar | 0,498 | Low HDI | Africa | Eastern Africa |
| Malawi | 0,414 | Low HDI | Africa | Eastern Africa |
| Malaysia | 0,773 | High HDI | Asia | South-Eastern Asia |
| Maldives | 0,698 | Medium HDI | Asia | Southern Asia |
| Mali | 0,407 | Low HDI | Africa | Western Africa |
| Malta | 0,829 | Very high HDI | Europe | Southern Europe |
| Mauritania | 0,487 | Low HDI | Africa | Western Africa |
| Mauritius | 0,771 | High HDI | Africa | Eastern Africa |
| Mexico | 0,756 | High HDI | Latin America and the Caribbean | Central America |
| Micronesia (Federated States of) | 0,630 | Medium HDI | Oceania | Micronesia |
| Moldova (Republic of) | 0,663 | Medium HDI | Europe | Eastern Europe |
| Mongolia | 0,698 | Medium HDI | Asia | Eastern Asia |
| Montenegro | 0,789 | High HDI | Europe | Southern Europe |
| Morocco | 0,617 | Medium HDI | Africa | Northern Africa |
| Mozambique | 0,393 | Low HDI | Africa | Eastern Africa |
| Myanmar | 0,524 | Low HDI | Asia | South-Eastern Asia |
| Namibia | 0,624 | Medium HDI | Africa | Southern Africa |
| Nepal | 0,540 | Low HDI | Asia | Southern Asia |
| Netherlands | 0,915 | Very high HDI | Europe | Western Europe |
| New Zealand | 0,910 | Very high HDI | Oceania | Australia and New Zealand |
| Nicaragua | 0,614 | Medium HDI | Latin America and the Caribbean | Central America |
| Niger | 0,337 | Low HDI | Africa | Western Africa |
| Nigeria | 0,504 | Low HDI | Africa | Western Africa |
| Norway | 0,944 | Very high HDI | Europe | Northern Europe |
| Oman | 0,783 | High HDI | Asia | Western Asia |
| Pakistan | 0,537 | Low HDI | Asia | Southern Asia |
| Palau | 0,775 | High HDI | Oceania | Micronesia |
| Palestine, State of | 0,686 | Medium HDI | Asia | Western Asia |
| Panama | 0,765 | High HDI | Latin America and the Caribbean | Central America |
| Papua New Guinea | 0,491 | Low HDI | Oceania | Melanesia |
| Paraguay | 0,676 | Medium HDI | Latin America and the Caribbean | South America |
| Peru | 0,737 | High HDI | Latin America and the Caribbean | South America |
| Philippines | 0,660 | Medium HDI | Asia | South-Eastern Asia |
| Poland | 0,834 | Very high HDI | Europe | Eastern Europe |
| Portugal | 0,822 | Very high HDI | Europe | Southern Europe |
| Qatar | 0,851 | Very high HDI | Asia | Western Asia |
| Romania | 0,785 | High HDI | Europe | Eastern Europe |
| Russian Federation | 0,778 | High HDI | Europe | Eastern Europe |
| Rwanda | 0,506 | Low HDI | Africa | Eastern Africa |
| Saint Kitts and Nevis | 0,750 | High HDI | Latin America and the Caribbean | Caribbean |
| Saint Lucia | 0,714 | High HDI | Latin America and the Caribbean | Caribbean |
| Saint Vincent and the Grenadines | 0,719 | High HDI | Latin America and the Caribbean | Caribbean |
| Samoa | 0,694 | Medium HDI | Oceania | Polynesia |
| Sao Tome and Principe | 0,558 | Medium HDI | Africa | Middle Africa |
| Saudi Arabia | 0,836 | Very high HDI | Asia | Western Asia |
| Senegal | 0,485 | Low HDI | Africa | Western Africa |
| Serbia | 0,745 | High HDI | Europe | Southern Europe |
| Seychelles | 0,756 | High HDI | Africa | Eastern Africa |
| Sierra Leone | 0,374 | Low HDI | Africa | Western Africa |
| Singapore | 0,901 | Very high HDI | Asia | South-Eastern Asia |
| Slovakia | 0,830 | Very high HDI | Europe | Eastern Europe |
| Slovenia | 0,874 | Very high HDI | Europe | Southern Europe |
| Solomon Islands | 0,491 | Low HDI | Oceania | Melanesia |
| South Africa | 0,658 | Medium HDI | Africa | Southern Africa |
| Spain | 0,869 | Very high HDI | Europe | Southern Europe |
| Sri Lanka | 0,750 | High HDI | Asia | Southern Asia |
| Sudan | 0,473 | Low HDI | Africa | Northern Africa |
| Suriname | 0,705 | High HDI | Latin America and the Caribbean | South America |
| Swaziland | 0,530 | Low HDI | Africa | Southern Africa |
| Sweden | 0,898 | Very high HDI | Europe | Northern Europe |
| Switzerland | 0,917 | Very high HDI | Europe | Western Europe |
| Syrian Arab Republic | 0,658 | Medium HDI | Asia | Western Asia |
| Taiwan | - | High HDI | Asia | Eastern Asia |
| Tajikistan | 0,607 | Medium HDI | Asia | Central Asia |
| Tanzania (United Republic of) | 0,488 | Low HDI | Africa | Eastern Africa |
| Thailand | 0,722 | High HDI | Asia | South-Eastern Asia |
| The former Yugoslav Republic of Macedonia | 0,732 | High HDI | Europe | Southern Europe |
| Timor Leste | 0,620 | Medium HDI | Asia | South-Eastern Asia |
| Togo | 0,473 | Low HDI | Africa | Western Africa |
| Tonga | 0,705 | High HDI | Oceania | Polynesia |
| Trinidad and Tobago | 0,766 | High HDI | Latin America and the Caribbean | Caribbean |
| Tunisia | 0,721 | High HDI | Africa | Northern Africa |
| Turkey | 0,759 | High HDI | Asia | Western Asia |
| Turkmenistan | 0,698 | Medium HDI | Asia | Central Asia |
| Uganda | 0,484 | Low HDI | Africa | Eastern Africa |
| Ukraine | 0,734 | High HDI | Europe | Eastern Europe |
| United Arab Emirates | 0,827 | Very high HDI | Asia | Western Asia |
| United Kingdom | 0,892 | Very high HDI | Europe | Northern Europe |
| United States | 0,914 | Very high HDI | Northern America | Northern America |
| Uruguay | 0,790 | High HDI | Latin America and the Caribbean | South America |
| Uzbekistan | 0,661 | Medium HDI | Asia | Central Asia |
| Vanuatu | 0,616 | Medium HDI | Oceania | Melanesia |
| Venezuela (Bolivarian Republic of) | 0,764 | High HDI | Latin America and the Caribbean | South America |
| Viet Nam | 0,638 | Medium HDI | Asia | South-Eastern Asia |
| Yemen | 0,500 | Low HDI | Asia | Western Asia |
| Zambia | 0,561 | Medium HDI | Africa | Eastern Africa |
| Zimbabwe | 0,492 | Low HDI | Africa | Eastern Africa |

* No document signed by Somalia, Democratic People's Republic of Korea, Holy See, Monaco, San Marino, Cook Islands, Marshall Islands, Nauru and Niue has been identified.
